# Plasma Phosphorylated Tau 217 and Aβ42/40 to Predict Early Brain Aβ Accumulation in People Without Cognitive Impairment

**DOI:** 10.1001/jamaneurol.2024.2619

**Published:** 2024-07-28

**Authors:** Shorena Janelidze, Nicolas R. Barthélemy, Gemma Salvadó, Suzanne E. Schindler, Sebastian Palmqvist, Niklas Mattsson-Carlgren, Joel B. Braunstein, Vitaliy Ovod, James G. Bollinger, Yingxin He, Yan Li, Cyrus A. Raji, John C. Morris, David M. Holtzman, Nicholas J. Ashton, Kaj Blennow, Erik Stomrud, Randall J. Bateman, Oskar Hansson

**Affiliations:** 1 Clinical Memory Research Unit, Department of Clinical Sciences Malmö, Lund University, Lund, Sweden; 2 Department of Neurology, Washington University School of Medicine in St Louis, St Louis, Missouri; 3 The Tracy Family SILQ Center, St Louis, Missouri; 4 The Knight ADRC, Washington University School of Medicine, St Louis, Missouri; 5 Memory Clinic, Skåne University Hospital, Malmö, Sweden; 6 Department of Neurology, Skåne University Hospital, Lund University, Lund, Sweden; 7 Wallenberg Center for Molecular Medicine, Lund University, Lund, Sweden; 8 C_2_N Diagnostics, St Louis, Missouri; 9 Department of Radiology and Neurology, Washington University in St Louis, St Louis, Missouri; 10 Department of Neurology, Hope Center for Neurological Disorders, Knight ADRC, Washington University in St Louis, St Louis, Missouri; 11 Institute of Neuroscience and Physiology, The Sahlgrenska Academy at University of Gothenburg, Mölndal, Sweden; 12 Wallenberg Centre for Molecular Medicine, University of Gothenburg, Gothenburg, Sweden; 13 King’s College London, Institute of Psychiatry, Psychology and Neuroscience, Maurice Wohl Institute Clinical Neuroscience Institute, London, United Kingdom; 14 NIHR Biomedical Research Centre for Mental Health and Biomedical Research Unit for Dementia at South London and Maudsley NHS Foundation, London, United Kingdom; 15 Paris Brain Institute, ICM, Pitié-Salpêtrière Hospital, Sorbonne University, Paris, France; 16 Clinical Neurochemistry Lab, Sahlgrenska University Hospital, Mölndal, Sweden

## Abstract

**Importance:**

Phase 3 trials of successful antiamyloid therapies in Alzheimer disease (AD) have demonstrated improved clinical efficacy in people with less severe disease. Plasma biomarkers will be essential for efficient screening of participants in future primary prevention clinical trials testing antiamyloid therapies in cognitively unimpaired (CU) individuals with initially low brain β-amyloid (Aβ) levels who are at high risk of accumulating Aβ.

**Objective:**

To investigate if combining plasma biomarkers could be useful in predicting subsequent development of Aβ pathology in CU individuals with subthreshold brain Aβ levels (defined as Aβ levels <40 Centiloids) at baseline.

**Design, Setting, and Participants:**

This was a longitudinal study including Swedish BioFINDER-2 (enrollment 2017-2022) and replication in 2 independent cohorts, the Knight Alzheimer Disease Research Center (Knight ADRC; enrollment 1988 and 2019) and Swedish BioFINDER-1 (enrollment 2009-2015). Included for analysis was a convenience sample of CU individuals with baseline plasma phosphorylated tau 217 (p-tau217) and Aβ42/40 assessments and Aβ assessments with positron emission tomography (Aβ-PET) or cerebrospinal fluid (CSF) Aβ42/40. Data were analyzed between April 2023 and May 2024.

**Exposures:**

Baseline plasma levels of Aβ42/40, p-tau217, the ratio of p-tau217 to nonphosphorylated tau (%p-tau217), p-tau231, and glial fibrillary acidic protein (GFAP).

**Main Outcomes and Measures:**

Cross-sectional and longitudinal PET and CSF measures of brain Aβ pathology.

**Results:**

This study included 495 (BioFINDER-2), 283 (Knight ADRC), and 205 (BioFINDER-1) CU participants. In BioFINDER-2, the mean (SD) age was 65.7 (14.4) with 261 females (52.7%). When detecting abnormal CSF Aβ-status, a combination of plasma %p-tau217 and Aβ42/40 showed better performance (area under the curve = 0.949; 95% CI, 0.929-0.970; *P* <.02) than individual biomarkers. In CU participants with subthreshold baseline Aβ-PET, baseline plasma %p-tau217 and Aβ42/40 levels were significantly associated with baseline Aβ-PET (n = 384) and increases in Aβ-PET over time (n = 224). Associations of plasma %p-tau217 and Aβ42/40 and their interaction with baseline Aβ-PET (%p-tau217: β = 2.77; 95% CI, 1.84-3.70; Aβ42/40: β = −1.64; 95% CI, −2.53 to −0.75; %p-tau217 × Aβ42/40: β = −2.14; 95% CI, −2.79 to −1.49; *P* < .001) and longitudinal Aβ-PET (%p-tau217: β = 0.67; 95% CI, 0.48-0.87; Aβ42/40: β = −0.33; 95% CI, −0.51 to −0.15; %p-tau217 × Aβ42/40: β = −0.31; 95% CI, −0.44 to −0.18; *P* < .001) were also significant in the models combining the 2 baseline biomarkers as predictors. Similarly, baseline plasma p-tau217 and Aβ42/40 were independently associated with longitudinal Aβ-PET in Knight ADRC (%p-tau217: β = 0.71; 95% CI, 0.26-1.16; *P* = .002; Aβ42/40: β = −0.74; 95% CI, −1.26 to −0.22; *P* = .006) and longitudinal CSF Aβ42/40 in BioFINDER-1 (p-tau217: β = −0.0003; 95% CI, −0.0004 to −0.0001; *P* = .01; Aβ42/40: β = 0.0004; 95% CI, 0.0002-0.0006; *P* < .001) in CU participants with subthreshold Aβ levels at baseline. Plasma p-tau231 and GFAP did not provide any clear independent value.

**Conclusions and Relevance:**

Results of this cohort study suggest that combining plasma p-tau217and Aβ42/40 levels could be useful for predicting development of Aβ pathology in people with early stages of subthreshold Aβ accumulation. These biomarkers might thus facilitate screening of participants for future primary prevention trials.

## Key Points

**Question:**

Which plasma biomarkers are useful to predict future development of Alzheimer disease (AD)–related β-amyloid (Aβ) pathology in cognitively unimpaired individuals (CU) with low brain Aβ levels?

**Findings:**

In this cohort study, which included CU individuals from 3 independent cohorts (the Swedish BioFINDER-2 study, Knight Alzheimer Disease Research Center, and the Swedish BioFINDER-1 study), a combination of baseline plasma phosphorylated tau 217 (p-tau217) and Aβ42/40 better predicted longitudinal changes in brain Aβ load than individual biomarkers in 514 participants with low brain Aβ levels at baseline.

**Meaning:**

Blood tests combining p-tau217 and Aβ42/40 levels could be useful for screening CU participants with early, subthreshold stages of brain Aβ pathology for future primary prevention trials in AD.

## Teaser

This cohort study investigates the utility of plasma markers phosphorylated tau 217 (p-tau217) and β-amyloid 42/40 (Aβ42/40) to predict development of Alzheimer disease–related Aβ pathology in cognitively unimpaired individuals with low brain Aβ levels.

## Introduction

In the last 2 years, 2 β-amyloid (Aβ)–clearing antibodies, aducanumab and lecanemab, have been approved by the US Food and Drug Administration for treatment of early symptomatic Alzheimer disease (AD),^1,2^ and another Aβ-targeting antibody, donanemab, has met the primary and secondary cognitive end points in a recently completed phase 3 randomized clinical trial.^3^ Data from the donanemab and lecanemab trials indicated that these disease-modifying therapies are more efficacious in people with less severe disease, ie, in those with lower levels of abnormal tau by positron emission tomography (PET).^3,4^ Thus, it is likely that future clinical trials will focus on early preclinical AD^5^ and ultimately move toward a primary prevention design defined as anti-Aβ treatment of individuals who do not yet have established Aβ pathology.^6^ To overcome challenges related to the enrollment of participants in primary prevention trials, there is a need for inexpensive and accessible blood-based biomarkers that could efficiently select individuals who still have low brain Aβ levels but are at a high risk of accumulating Aβ pathology in the future. Plasma levels of Aβ42/40 and phosphorylated tau (p-tau) quantified using novel highly sensitive methods appear to accurately reflect AD neuropathological changes, ie, accumulation of Aβ plaques and neurofibrillary tangles.^7,8,9,10,11,12,13^ Interestingly, increases in the levels of soluble p-tau (in plasma and cerebrospinal fluid [CSF]) are strongly associated with brain Aβ pathology, and both plasma Aβ42/40 and p-tau levels individually show high performance when differentiating abnormal from normal Aβ-PET status.^12,13,14^ Two previous studies^15,16^ have reported improved detection of Aβ positivity when using models combining plasma Aβ42/40 and p-tau217 (tau phosphorylated at threonine 217) levels or %p-tau217 (the ratio of p-tau217 to nonphosphorylated tau) in cognitively unimpaired (CU) participants. Here, we aimed to explore the utility of blood-based biomarkers a step further, namely, for selection of participants in future primary prevention clinical trials. To this end, we examined the ability of plasma %p-tau217 and Aβ42/40 levels, as well as their combination, to predict the development of Aβ pathology over time in CU individuals with subthreshold brain Aβ levels from the Swedish BioFINDER-2 study. In addition, we investigated the potential benefit of adding plasma p-tau231 and glial fibrillary acidic protein (GFAP), both of which have previously been linked to the early stages of brain Aβ accumulation.^17,18,19,20,21,22^ Finally, we validated BioFINDER-2 findings in 2 independent cohorts, the Knight Alzheimer Disease Research Center (Knight ADRC) and the Swedish BioFINDER-1 study.

## Methods

### Participants

All participants from the Swedish BioFINDER-2 (NCT03174938^7^) and the Swedish BioFINDER-1 (NCT01208675^23^) studies provided written informed consent. In the Knight ADRC, written informed consent was obtained from each participant or their legally authorized representative when appropriate. Ethical approval was given by the Swedish Ethical Review Authority and the Washington University Human Research Protection Office. Race and ethnicity information was not collected in the BioFINDER studies. The majority of the Knight ADRC participants self-identified as non-Hispanic White. All CU participants who had plasma p-tau217 and Aβ42/40 assessments and either Aβ-PET (BioFINDER-2, Knight ADRC) as described in the Aβ-PET imaging and processing section or CSF Aβ42/40 (BioFINDER-1 where longitudinal Aβ-PET is not available) were included. Further details of inclusion/exclusion criteria have been previously described^7,24,25^ and are also provided in the eMethods in Supplement 1. This study followed the Standards for Reporting of Diagnostic Accuracy (STARD) reporting guidelines.

### Plasma and CSF Analysis

In the BioFINDER-2 and Knight ADRC cohorts, plasma levels of Aβ42, Aβ40, and %p-tau217 were measured using liquid chromatography–tandem mass spectrometry at the Department of Neurology, Washington University School of Medicine, or at C_2_N Diagnostics (Precivity tests of Aβ42, Aβ40 in the Knight ADRC).^8,26,27^ In the BioFINDER-1 study, plasma concentration of p-tau217 was determined at Lund University using an immunoassay developed by Lilly Research Laboratories.^15,28^ Plasma levels of p-tau231 and GFAP were measured using in-house Simoa immunoassay developed at the University of Gothenburg and commercially available Simoa Discovery immunoassay (Quanterix), respectively.^17,22^ CSF levels of Aβ40 and Aβ42 were assessed using Roche Elecsys immunoassay and NeuroToolKit, respectively, or Lumipulse G (Fujirebio) immunoassays. CSF Aβ status (negative/positive) was determined using the CSF Aβ42/40 ratio based on previously described cutoffs.^29,30,31,32^ Plasma and CSF analysis are further described in the eMethods in Supplement 1. All samples were analyzed by staff blinded to the clinical data.

### Aβ-PET Imaging and Processing

Aβ-PET imaging was performed using [^18^F]-flutemetamol in the BioFINDER-2 study and either [^18^F]-AV45 or carbon 11–labeled Pittsburgh compound B (PiB) in the Knight ADRC cohort^7,24^ (eMethods in Supplement 1). BioFINDER-2 study participants underwent their first Aβ-PET scans at baseline visit within 1 year of blood collection. In the Knight ADRC cohort, Aβ-PET scans performed with the same tracer within each individual were included in the longitudinal analysis and only if the first scan was performed not more than 5 years before blood collection.

Mean cortical standardized uptake value ratio values for [^18^F]flutemetamol, PiB, and ^18^F-AV45 were then transformed to Centiloid (CL) units^33^ for better comparability within this study, and with other studies. In the main analysis, similar to the A45 trial of the AHEAD 3-45 study,^5^ we used a cutoff of less than 40 CL to define subthreshold Aβ levels and identify CU individuals who did not have elevated brain Aβ levels at the visit closest to plasma collection. We also performed sensitivity analyses for 20-CL and 12-CL thresholds.^34,35,36^ To categorize study participants as Aβ accumulators or nonaccumulators, we applied a previously defined threshold of greater than 3.0 CL per year.^37^

### Statistical Analysis

The programming language R, version 4.1.2 (R Project for Statistical Computing), was used for statistical analysis. Discriminative accuracies of plasma biomarkers were assessed and compared using logistic regression models, receiver operating characteristic (ROC) curve analysis, and the DeLong test. Group differences were examined using Wilcoxon rank sum test. Associations between plasma biomarkers and Aβ-PET at baseline were studied using linear regression models adjusting for age and sex. We first tested if baseline levels of %p-tau217 or Aβ42/40 were related to baseline Aβ-PET in the models including one of the biomarkers as predictors. Next, baseline %p-tau217 and Aβ42/40 levels were combined as predictors in the same model to determine if these biomarkers were independently associated with Aβ-PET. We also tested models that included the interaction between %p-tau217 and Aβ42/40. Finally, we included either baseline p-tau231 or GFAP together with %p-tau217 and Aβ42/40 in the same models to assess if either p-tau231 or GFAP correlated with baseline Aβ-PET when accounting for the effects of plasma %p-tau217 and Aβ42/40 levels. For associations with longitudinal Aβ measures, we first derived individual slopes of Aβ-PET in the BioFINDER-2 study and CSF Aβ42/40 in the BioFINDER-1 study from linear mixed-effects models including longitudinal Aβ-PET or CSF Aβ42/40 as outcome and time (years since baseline) as predictor with random slopes and intercepts. Because a large majority of the participants in the Knight ADRC only had 2 Aβ-PET scans, slopes of Aβ-PET in this cohort were derived using participant-level linear regression models with longitudinal Aβ-PET as the outcome and time since plasma collection as the predictor. Associations between baseline levels of p-tau217 or Aβ42/40 (individually or in combination) with Aβ-PET or CSF Aβ42/40 slopes were tested using linear regression models adjusting for age and sex. For goodness of fit, we reported adjusted coefficient of determination (adjusted *R*^2^) and Akaike information criterion (AIC). Models were considered significantly different if change in AIC was less than 2. In regression analysis of continuous Aβ-PET data, plasma biomarker measures were first log10-transformed to better fit the normal distribution and then standardized to the CU population that was Aβ negative (reference) for increased interpretability. All *P* values were 2-sided, and *P* <.05 was considered significant. Data were analyzed between April 2023 and May 2024.

## Results

### The BioFINDER-2 Study

#### Participants

The BioFINDER-2 cohort included 495 CU participants (Table 1); the mean (SD) age of the cohort was 65.7 (14.4) years, of whom 261 (52.7%) were female and 234 (47.3%) were male. Cross-sectional CSF Aβ42/40 and Aβ-PET data were available for 492 and 449 participants, respectively (all 495 participants had either CSF Aβ42/40 or Aβ-PET). Longitudinal Aβ-PET was available in 224 CU participants with subthreshold baseline Aβ levels (Aβ-PET <40 CL; 144 and 80 participants with 2 and 3 scans, respectively; the mean [SD] time between the first and last scan was 2.7 [1.0] years) (eTable 1 in Supplement 1).

**Table 1.** Demographic and Clinical Characteristics, the Swedish BioFINDER-2 Study

Characteristic	Cognitively unimpaired	Cognitively unimpaired Aβ-PET<40 CL
No.	495	384
Age, median (IQR), y	67.8 (55.8-77.3)	64.6 (53.9-76.0)
Sex, No. (%)		
Female	261 (52.7)	203 (52.9)
Male	234 (47.3)	181 (47.1)
MMSE, median (IQR)	29.0 (28.0-30.0)	29.0 (28.0-30.0)
Education, median (IQR), y^a^	12.0 (11.0-15.0)	13.0 (11.0-15.0)
*APOE* ε4 positivity, No. (%)^a^	170 (34.3%)	130 (33.9%)
CSF Aβ42/40 positivity, No. (%)	136 (27.6%)	63 (16.4%)
Plasma %p-tau217, median (IQR)	0.744 (0.604-1.05)	0.705 (0.590-0.868)
Plasma Aβ42/40, median (IQR)	0.119 (0.111-0.126)	0.120 (0.113-0.127)
Plasma p-tau231, median (IQR),^a^ pg/mL	5.2 (3.9-7.1)	4.8 (3.8-6.3)
Plasma GFAP, median (IQR),^a^ pg/mL	106.0 (69.9-160.0)	95.5 (64.7-141.0)

Abbreviations: Aβ, β amyloid; *APOE*, apolipoprotein E; CL, Centiloid; CSF, cerebrospinal fluid; GFAP, glial fibrillary acidic protein; MMSE, Mini Mental State Examination; PET, positron emission tomography; p-tau, phosphorylated tau; %p-tau217, ratio of phosphorylated tau 217 (p-tau217) to nonphosphorylated tau.

^a^ Education, *APOE* ε4, plasma p-tau231, and GFAP were missing for 2, 91, 79 and 69 participants, respectively.

#### Associations Between Plasma Biomarkers and Brain Aβ Status at Baseline

We first studied the ability of the plasma biomarkers to identify CU individuals with elevated brain Aβ level according to either CSF Aβ42/40 or Aβ-PET. When differentiating normal vs abnormal CSF Aβ42/40, a combination of plasma %p-tau217 and Aβ42/40 had significantly higher AUCs (0.949; 95% CI 0.929-0.970) than %p-tau217 by itself (0.924; 95% CI, 0.894-0.954; *P* = .02) or Aβ42/40 by itself (0.849; 95% CI, 0.810-0.887; *P* <.001) (eFigure 1 and eTable 2 in Supplement 1).

However, plasma %p-tau217 alone had very high AUC (0.969-0.976) for discriminating elevated Aβ-PET status, and no further improvements were seen when combining plasma %p-tau 217 and Aβ42/40 measures (eTable 2 in Supplement 1). In addition, we did not find any improvement in AUCs when adding plasma p-tau231 or GFAP to the combination of plasma %p-tau217 and Aβ42/40 measures (eTables 3 and 4 in Supplement 1).

#### Associations Between Baseline Plasma Biomarkers and Continuous Measures of Baseline and Longitudinal Aβ-PET

Identifying CU individuals with initially low brain Aβ levels who are at increased risk of accumulating Aβ pathology would be critical for selection of the participants in primary prevention clinical trials. We therefore focused on CU participants with subthreshold baseline Aβ levels (Aβ-PET <40 CL), and first performed cross-sectional analyses studying the associations between plasma biomarkers and continuous measures of Aβ-PET at baseline, followed by longitudinal analyses investigating associations between baseline plasma biomarkers and changes in Aβ-PET over time.

**Figure 1.**
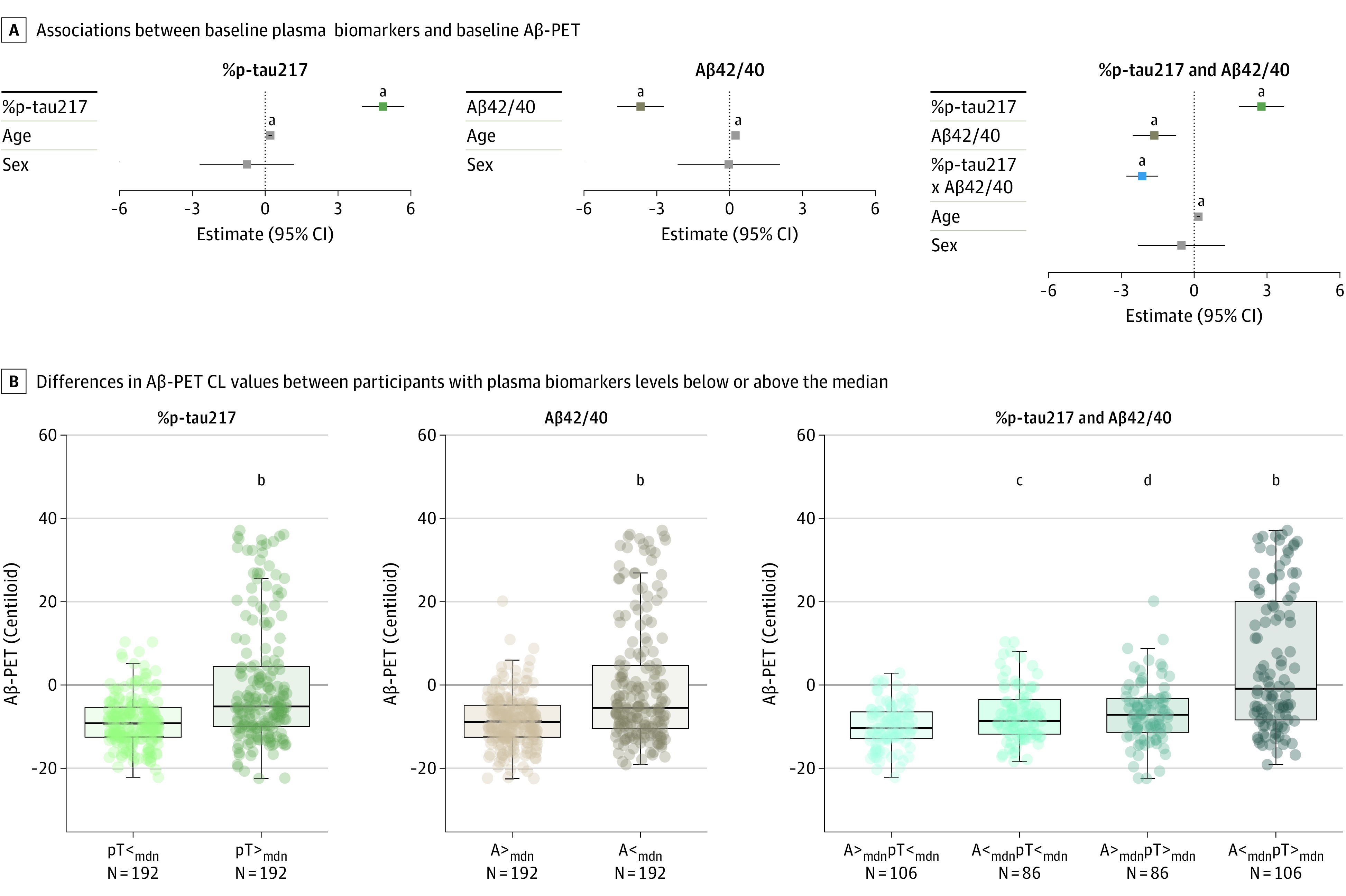
Associations Between Plasma Biomarkers and β-Amyloid Positron Emission Tomography (Aβ-PET) at Baseline in Cognitively Unimpaired (CU) Participants With Subthreshold Baseline Aβ-PET A, Estimates and 95% CIs from linear regression models including continuous measures of the ratio of phosphorylated tau 217 (p-tau217) to non–p-tau (%p-tau217), Aβ42/40 or %p-tau217, Aβ42/40 and their interaction, as well as age and sex as predictors and Aβ-PET as outcome. Log-transformed and *z*-scored plasma biomarkers were used in all regression models. Aβ-PET status was defined by a threshold of 40 Centiloids (CL). B, Differences in Aβ-PET Centiloid values between participants with plasma biomarkers levels below or above the median (mdn). A indicates Aβ42/40, pT, %p-tau217. ^a^*P* <.001. ^b^*P* <.0001. ^c^*P* <.05. ^d^*P* <.01.

In the cross-sectional analysis (Figure 1 and Table 2), linear regression models including one of the plasma biomarkers as the predictor showed that both higher baseline %p-tau217 (model 1, β = 4.85; 95% CI, 3.98-5.72; *P* < .001; adjusted *R*^2^ = 0.34) and lower Aβ42/40 (model 2, Aβ42/40: β = −3.67; 95% CI, −4.63 to −2.71; *P* < .001; adjusted *R*^2^ = 0.25) were associated with higher Aβ-PET Centiloids in CU participants with subthreshold baseline Aβ when assessed in different models. Associations with Aβ-PET CL were also significant for both %p-tau217 and Aβ42/40 in the models combining the 2 biomarkers as predictors (model 3, %p-tau217: β = 2.77; 95% CI, 1.84-3.70; Aβ42/40: β = −1.64; 95% CI, −2.53 to −0.75; *P* < .001), where we found a significant effect of %p-tau217 and Aβ42/40 interaction (model 3, β = −2.14; 95% CI, −2.79 to −1.49; *P* < .001; adjusted *R*^2^ = 0.45) (Figure 1A). When plasma biomarkers were dichotomized at the median into high vs low categories, the largest increase in Aβ-PET Centiloids (compared with the group high Aβ42/40, low %p-tau217) was observed in the group low Aβ42/40 and high %p-tau217 (Figure 1B). Similarly, these associations were also significant when Aβ-PET thresholds of 20 CL or 12 CL were applied to identify those with low baseline Aβ-PET levels to be included in the analyses (Table 2). However, the effect sizes decreased at lower CL thresholds and %p-tau217 × Aβ42/40 interaction was no longer significant for less than 12 CL. Neither baseline plasma p-tau231 nor GFAP were independently associated with baseline Aβ-PET CL in the multivariate models also including baseline plasma %p-tau217 and Aβ42/40 as predictors (eTable 5 in Supplement 1), and therefore, these biomarkers were excluded from the longitudinal analysis.

**Table 2.** Associations of Baseline Ratio of Phosphorylated Tau 217 (p-Tau217) to Nonphosphorylated Tau (%p-Tau217) and β-Amyloid 42/40 (Aβ42/40) With Baseline Aβ Positron Emission Tomography (Aβ-PET) in Cognitively Unimpaired (CU) Participants With Subthreshold Baseline Aβ-PET, the Swedish BioFINDER-2 Study^a^

Variable	Model		%p-tau217		Aβ42/40		%p-tau217 × Aβ42/40	
	Adjusted R2	AIC	β (95% CI)	*P* value	β (95% CI)	*P* value	β (95% CI)	*P* value
**<40 CL (n = 384)**								
Model 1, %p-tau217	0.343	2838.10	4.85 (3.98 to 5.72)	1.4 × 10^−24^	NA	NA	NA	NA
Model 2, Aβ42/40	0.247	2890.74	NA	NA	−3.67 (−4.63 to −2.71)	3.8 × 10^−13^	NA	NA
Model 3, %p-tau217 and Aβ42/40	0.453	2769.78	2.77 (1.84 to 3.70)	9.9 × 10^−9^	−1.64 (−2.53 to −0.75)	4.0× 10^−4^	−2.14 (−2.79 to −1.49)	3.9 × 10^−10^
**<20 CL (n = 356)**								
Model 1, %p-tau217	0.204	2364.64	1.72 (1.04 to 2.40)	1.0 × 10^−6^	NA	NA	NA	NA
Model 2, Aβ42/40	0.184	2373.45	NA	NA	−1.36 (−2.03 to −0.68)	9.5 × 10^−5^	NA	NA
Model 3, %p-tau217 and Aβ42/40	0.244	2348.41	1.20 (0.49 to 1.92)	.001	−1.00 (−1.67 to −0.34)	.003	−0.95 (−1.62 to −0.29)	.005
**<12 CL (n = 347)**								
Model 1, %p-tau217	0.168	2223.37	0.67 (0.03 to 1.31)	.04	NA	NA	NA	NA
Model 2, Aβ42/40	0.171	2222.14	NA	NA	−0.72 (−1.33 to −0.11)	.02	NA	NA
Model 3, %p-tau217 and Aβ42/40	0.176	2221.99	0.68 (0.02 to 1.35)	.04	−0.71 (−1.32 to −0.10)	.02	0.21 (−0.50 to 0.92)	.57

Abbreviations: AIC, Akaike information criterion; CL, Centiloid; NA, not applicable.

^a^ Data are from linear regression models including continuous measures of %p-tau217 (model 1), Aβ42/40 (model 2) or %p-tau217, Aβ42/40 and their interaction (model 3) as predictors and baseline Aβ-PET as outcome. The models included age and sex, as covariates.

**Figure 2.**
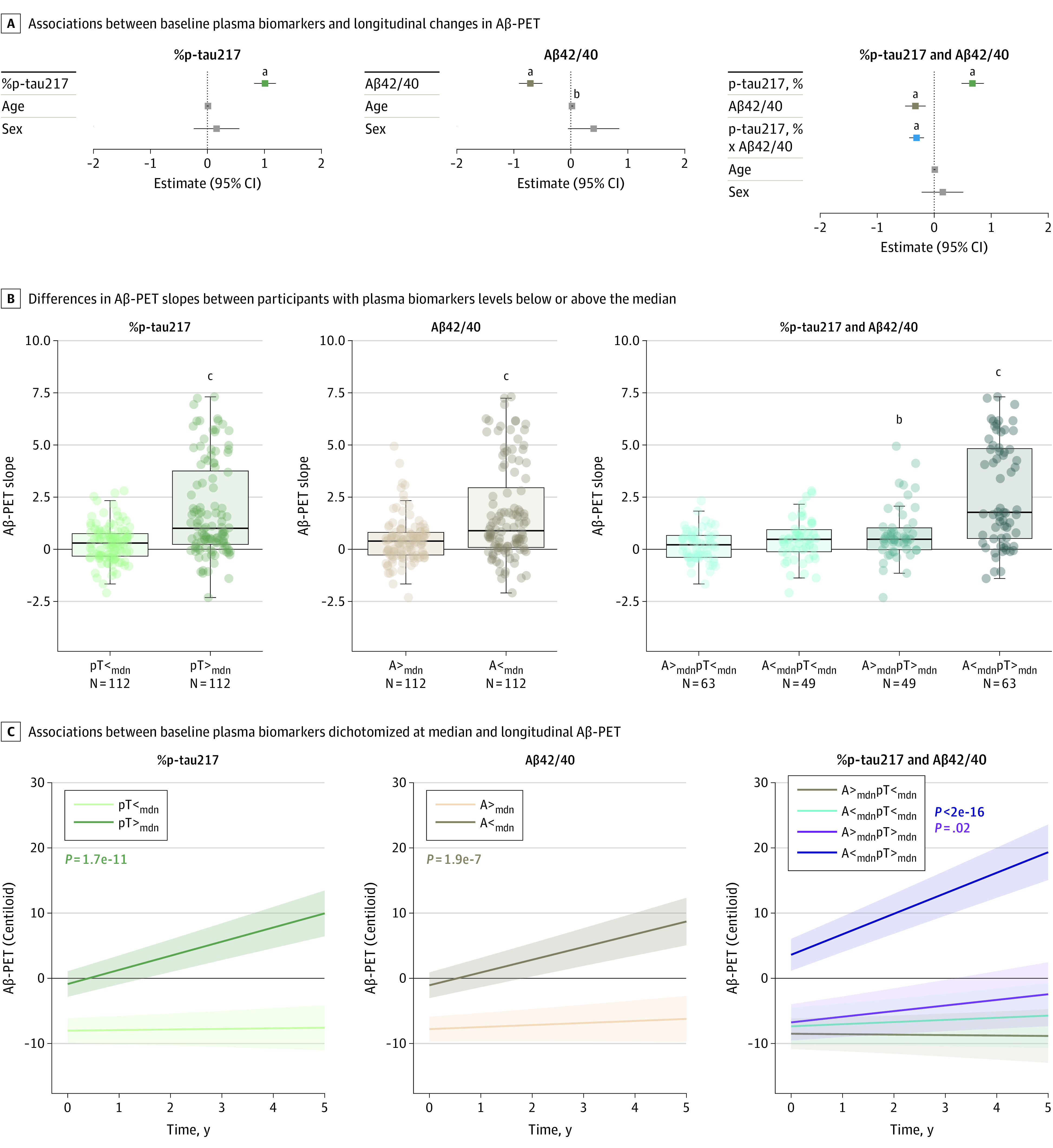
Associations Between Plasma Biomarkers and Longitudinal β-Amyloid Positron Emission Tomography (Aβ-PET) in Cognitively Unimpaired (CU) Participants With Subthreshold Baseline Aβ-PET A, Associations between baseline plasma biomarkers and longitudinal changes in Aβ-PET were tested using linear regression models including continuous measures of the ratio of phosphorylated tau 217 (p-tau217) to non–p-tau (%p-tau217), Aβ42/40 or %p-tau217, Aβ42/40 and their interaction, as well as age and sex as predictors and subject-specific slopes of Aβ-PET as outcome. Participant-specific slopes of Aβ-PET were derived from linear mixed-effects models including longitudinal Aβ-PET as outcome and time (years since baseline) as predictor. Log-transformed and *z*-scored plasma biomarkers were used in all regression models. Aβ-PET status was defined by a threshold of 40 Centiloids (CL). B, Differences in Aβ-PET slopes between participants with plasma biomarkers levels below or above the median (mdn). C, Associations between plasma biomarkers and longitudinal Aβ-PET were visualized using linear mixed-effects models with Aβ-PET as outcome and interaction between plasma biomarkers (dichotomized at mdn) and time as predictor adjusting for age and sex. The ggeffects package of R software was used to generate estimated values. A indicates Aβ42/40, pT, %p-tau217. ^a^*P* <.001. ^b^*P* <.05. ^c^*P* <.0001.

In linear regression models including 1 of the plasma biomarkers as predictor, higher baseline %p-tau217 (model 1, β = 1.01; 95% CI, 0.82-1.20; *P* < .001; adjusted *R*^2^ = 0.37) and lower Aβ42/40 (model 2, Aβ42/40: β = −0.71; 95% CI, −0.91 to −0.50; *P* < .001; adjusted *R*^2^ = 0.21) were both associated with higher participant-specific slopes of Aβ-PET (Figure 2 and Table 3). Furthermore, associations with longitudinal changes in Aβ-PET were significant for both %p-tau217 and Aβ42/40, and there was a significant %p-tau217 × Aβ42/40 interaction in the models combining the 2 biomarkers as predictors (model 3, %p-tau217: β = 0.67; 95% CI, 0.48-0.87; Aβ42/40: β = −0.33; 95% CI, −0.51 to −0.15; %p-tau217 × Aβ42/40: β = −0.31; 95% CI, −0.44 to −0.18; *P* < .001; adjusted *R*^2^ = 0.48). The group with low Aβ42/40 and high %p-tau217 again showed higher Aβ-PET slopes than the other groups (Figure 2B). Significant associations (with smaller effect sizes) were also seen when Aβ-PET thresholds of 20 CL or 12 CL were applied to determine Aβ-PET status at baseline (Table 3) or when adjusting for baseline Aβ-PET (eTable 6 in Supplement 1).

**Table 3.** Associations of Baseline Ratio of Phosphorylated Tau 217 (p-Tau217) to Nonphosphorylated Tau (%p-tau217) and β-Amyloid 42/40 (Aβ42/40) With Longitudinal Aβ Positron Emission Tomography (Aβ-PET) in Cognitively Unimpaired (CU) Participants With Subthreshold Baseline Aβ-PET, the Swedish BioFINDER-2 Study^a^

Variable	Model		%p-tau217		Aβ42/40		%p-tau217 × Aβ42/40	
	Adjusted *R*^2^	AIC	β (95% CI)	*P* value	β (95% CI)	*P* value	β (95% CI)	*P* value
**<40 CL (n = 224)**								
Model 1, %p-tau217	0.369	824.20	1.01 (0.82 to 1.20)	1.3 × 10^−21^	NA	NA	NA	NA
Model 2, Aβ42/40	0.210	874.33	NA	NA	−0.71 (−0.91 to −0.50)	9 × 10^−11^	NA	NA
Model 3, %p-tau217 and Aβ42/40	0.484	781.11	0.67 (0.48 to 0.87)	1.0 × 10^−10^	−0.33 (−0.51 to −0.15)	4.0× 10^−4^	−0.31 (−0.44 to −0.18)	3.7 × 10^−6^
**<20 CL (n = 209)**								
Model 1, %p-tau217	0.202	715.98	0.67 (0.47 to 0.86)	8.5 × 10^−11^	NA	NA	NA	NA
Model 2, Aβ42/40	0.082	745.12	NA	NA	−0.37 (−0.56 − 0.17)	2.0× 10^−4^	NA	NA
Model 3, %p-tau217 and Aβ42/40	0.307	688.30	0.53 (0.34 to 0.71)	5.6 × 10^−8^	−0.26 (−0.43 to −0.09)	.003	−0.42 (−0.60 to −0.24)	8.4 × 10^−6^
**<12 CL (n = 203)**								
Model 1, %p-tau217	0.135	646.28	0.47 (0.29 to 0.65)	4.5 × 10^−7^	NA	NA	NA	NA
Model 2, Aβ42/40	0.047	665.95	NA	NA	−0.22 (−0.39 to −0.05)	.013	NA	NA
Model 3, %p-tau217 and Aβ42/40	0.175	638.68	0.43 (0.26 to 0.61)	2.3 × 10^−6^	−0.19 (−0.35 to −0.03)	.02	−0.22 (−0.41 to −0.04)	.02

Abbreviations: AIC, Akaike information criterion; CL, Centiloid; NA, not applicable.

^a^ Data are from linear regression models including continuous measures of %p-tau217 (model 1), Aβ42/40 (model 2) or %p-tau217, Aβ42/40 and their interaction (model 3) to predict the participant-specific slopes of Aβ-PET. All models included age and sex as covariates. Participant-specific slopes of Aβ-PET were derived from linear mixed-effects models including longitudinal Aβ-PET as outcome and time (years since baseline) as predictor.

### Sensitivity Analysis

In addition to using slopes of Aβ-PET, we tested associations between the plasma biomarkers and longitudinal Aβ-PET with linear mixed-effects models, which showed similar results (Figure 2C and eTable 7 in Supplement 1). We also assessed the accuracy of plasma biomarkers to identify CU participants classified as accumulators or those who progressed to Aβ-PET positivity with specificity set to 90% because high specificity is essential for efficient screening in prevention trials. In the ROC curve analysis (eTables 8-9 in Supplement 1), including CU individuals with baseline Aβ-PET less than 40 CL, plasma %p-tau217 showed high AUCs (0.949-0.954) and sensitivities (83%-84%) for both outcomes. For lower Centiloid thresholds (<20 CL and <12 CL), AUCs of %p-tau217 remained high (0.871-0.931); however, there was a decrease in sensitivities (56%-80%). No significant increases in AUCs were seen when combining %p-tau217 and Aβ42/40 measures, which is likely due to low number of CU categorized as either accumulators (n = 11-32) or progressors (n = 9-12).

### Replication in 2 Independent Cohorts

#### Knight ADRC

We first replicated the BioFINDER-2 findings in 283 CU individuals (mean [SD] age, 67.9 [8.3] years) from the Knight ADRC cohort (eTable 10 in Supplement 1), of whom 151 (53.4%) were female and 132 (46.6%) were male. When detecting abnormal CSF Aβ42/40 at baseline, a combination of plasma %p-tau217 and Aβ42/40 again had a significantly higher AUC (0.944; 95% CI, 0.912-0.976) than %p-tau217 by itself (0.895; 95% CI, 0.845-0.946; *P* = .01) or Aβ42/40 by itself (0.855; 95% CI, 0.810-0.900; *P *<.001) (eFigure 1 and eTable 11 in Supplement 1). Similar to the BioFINDER-2 study, discriminative accuracies for Aβ-PET status at baseline were not different between models including %p-tau217 alone and those combining %p-tau 217 and Aβ42/40 measures (eTable 11 in Supplement 1). Furthermore, cross-sectional analysis including only CU participants with subthreshold (<40 CL) Aβ-PET (eTable 12 in Supplement 1) revealed significant associations between plasma %p-tau217 (model 1, β = 3.85; 95% CI, 2.64-5.05; *P* < .001; adjusted *R*^2^ = 0.15) or Aβ42/40 (model 2, β = −2.74; 95% CI, −4.18 to −1.29; *P* < .001; adjusted *R*^2^ = 0.06) and Aβ-PET (eTable 13 in Supplement 1). Plasma %p-tau217 and interaction between %p-tau217 and Aβ42/40 were also associated with Aβ-PET in the models combining the 2 biomarkers as predictors (model 3, %p-tau217: β = 2.17; 95% CI, 0.86-3.47; *P* = .001; %p-tau217 × Aβ42/40: β = −2.17; 95% CI, −3.11 to −1.23; *P* < .001; adjusted *R*^2^ = 0.23) (eFigure 2A and eTables 13 in Supplement 1). Longitudinal Aβ-PET was available for 85 CU individuals with subthreshold brain Aβ at the time of plasma collection in the Knight ADRC cohort (Aβ-PET <40 CL; 73 participants had 2 scans, 11 participants had 3 scans, and 1 participant had 4 scans). The mean (SD) time between the first and last scan was 5.0 (2.5) years. In this sample, both baseline plasma %p-tau217 and Aβ42/40 were also associated with higher participant-specific slopes of Aβ-PET in the models including 1 of the plasma biomarkers (model 1, %p-tau217: β = 0.81; 95% CI, 0.35-1.26; *P* < .001, adjusted *R*^2^ = 0.14; model 2, Aβ42/40: β = −0.87; 95% CI, −1.41 to −0.32; *P* = .002; adjusted *R*^2^ = 0.12) or both biomarkers (model 3, %p-tau217: β = 0.71; 95% CI, 0.26-1.16; *P* = .002; Aβ42/40: β = −0.74; 95% CI, −1.26 to −0.22; *P* = .006; adjusted *R*^2^ = 0.21) as predictors (eFigure 3A and eTable 13 in Supplement 1). When comparing with the group high Aβ42/40, low %p-tau217, the largest increases in Aβ-PET CL and Aβ-PET slopes were seen in the group low Aβ42/40, high %p-tau217 (eFigures 2B and 3B in Supplement 1). In the ROC curve analysis, %p-tau217 showed relatively high AUC (0.844) and sensitivity (79%) when differentiating accumulators (n = 14) from nonaccumulators (n = 71) with no improved performance of the model combining %p-tau217 and Aβ42/40 (eTable 14 in Supplement 1). Only 3 participants from the Knight ADRC progressed to Aβ-PET positivity during follow-up.

#### The BioFINDER-1 Study

Finally, we replicated the longitudinal findings in the BioFINDER-1 cohort using CSF Aβ42/40 as a measure of brain Aβ pathology. From BioFINDER-1, we included 205 individuals (mean [SD] age, 71.9 [5.4] years) with normal baseline CSF Aβ status (of whom 127 [62.0%] were female and 78 [38.0%] were male) who had longitudinal CSF Aβ42/40 data (mean [SD] time between the first and last LP was 4.8 [1.9] years) (eTable 12 in Supplement 1). In this cohort, higher baseline levels of plasma p-tau217 and lower plasma Aβ42/40 were associated with longitudinal decreases in CSF Aβ42/40 over time in the models including either of the plasma biomarkers as predictors (model 1, p-tau217: β = −0.0003; 95% CI, −0.0005 to −0.0001; *P* = .004, adjusted *R*^2^ = 0.04; model 2, Aβ42/40: β = 0.0005; 95% CI, 0.0003-0.0007; *P* < .001; adjusted *R*^2^ = 0.09) or both (model 3, p-tau217: β = −0.0003; 95% CI, −0.0004 to −0.0001; *P* = .012; Aβ42/40: β = 0.0004; 95% CI, 0.0002-0.0006; *P* < .001; adjusted *R*^2^ = 0.11) (eTable 15 in Supplement 1).

## Discussion

The main results of this cohort study suggest that a blood test measuring p-tau217 and Aβ42/40 could be useful for detection of early stages of brain Aβ deposition. First, we show that combining plasma %p-tau217 and Aβ42/40 improved classification of CSF Aβ-status compared with using these same individual biomarkers alone in CU from both the BioFINDER-2 and Knight ADRC cohorts. Next, we found that in CU participants with subthreshold baseline brain Aβ levels from the BioFINDER-2 study, plasma %p-tau217 level, and plasma Aβ42/40 level, as well as their interaction, were associated with Aβ-PET Centiloid values at baseline and, more importantly, with the increases in Aβ-PET over time. The group with higher than median %p-tau217 and lower than median Aβ42/40 had the highest baseline Aβ-PET and largest increase in Aβ-PET overtime compared with other groups (ie, Aβ42/40 >median, %p-tau217 <median; Aβ42/40 <median, %p-tau217 <median; Aβ42/40 >median, %p-tau217 >median)_._ The BioINDER-2 findings were replicated in the Knight ADRC and BioFINDER-1 cohorts, where baseline plasma p-tau217 and Aβ42/40 were independently associated with longitudinal changes in Aβ-PET and CSF Aβ42/40 in CU participants with subthreshold Aβ levels at baseline. Finally, we did not see any significant associations between plasma p-tau231 or GFAP and Aβ-PET levels when accounting for the effects of plasma %p-tau217 and Aβ42/40.

Altered plasma levels of Aβ42/40, different p-tau variants (in particular p-tau217 and p-tau231), and GFAP are increasingly recognized to reflect early Aβ deposition in preclinical and prodromal stages of AD.^12,13^ It is reasonable to assume that slow Aβ accumulation seen even in some CU individuals negative for Aβ-PET^38,39^ would also impact plasma biomarker concentrations. However, associations between plasma biomarkers and measures of Aβ pathology in this population are unexplored. Although 1 previous study^27^ reported that in CU individuals negative for Aβ-PET, abnormal baseline plasma Aβ42/40 was associated with 15-fold increased risk of progression to amyloid PET-positivity, no data are available, to the best of our knowledge, for other plasma biomarkers or biomarker combinations. Here, first using baseline data from the BioFINDER-2 and Knight ADRC cohorts, we show that in CU individuals with subthreshold Aβ-PET scans, plasma %p-tau217 and Aβ42/40 were both associated with higher Aβ-PET uptake. No significant associations with Aβ-PET were observed for either plasma p-tau231 or GFAP when they were added to the models already including plasma %p-tau217 and Aβ42/40. Thus, even though plasma levels of p-tau231 and GFAP are increased in response to Aβ pathology early in the disease course,^18,19,20,21,22^ our results suggest that they do not provide any added value as indicators of brain Aβ burden beyond the effects of plasma %p-tau217 and Aβ42/40.

One of the key findings of the present study, reproduced in both the BioFINDER-2 and Knight ADRC cohorts, is that in CU individuals with subthreshold baseline Aβ-PET, baseline plasma %p-tau217, and baseline plasma Aβ42/40 were significant predictors of longitudinal increases in Aβ-PET when combined in the same model. Furthermore, similar results were seen in the BioFINDER-1 study when using CSF Aβ42/40 as a measure of longitudinal brain Aβ accumulation. In addition, we observed a significant interaction effect between %p-tau217 and Aβ42/40 in the BioFINDER-2 study, suggesting that the effects %p-tau217 on slopes of Aβ-PET were dependent on the value of Aβ42/40. The associations between baseline plasma biomarkers and longitudinal increases in Aβ-PET were significant even when adjusting for the effects of baseline Aβ-PET, which in earlier work has been shown to relate to accelerated Aβ-PET accumulation.^38,40^ Collectively, these data have important implications for future clinical trials in AD especially considering that in CU individuals increasing Aβ, although still in negative range, has been linked to subsequent tau deposition and worsening of cognitive function.^38,40,41^ After their success in slowing cognitive decline in symptomatic AD, lecanemab and donanemab are currently being tested in secondary prevention trials such as AHEAD 3-45 and Trailblazer-ALZ3, respectively, that enroll asymptomatic people with biomarker-evidence of brain Aβ pathology. However, it is likely that the greatest effects of Aβ-lowering treatments would be achieved through primary prevention in individuals with normal brain Aβ levels who are at a high risk of accumulating Aβ pathology. The results of the present study highlight the potential utility of plasma %p-tau217 and Aβ42/40 for identifying individuals to be included in such trials. Going forward, it will be important to define how these biomarkers should be implemented in the screening process to optimize participant enrollment. Although our data from the ROC curve analysis indicated high performance of %p-tau217 when identifying accumulators or progressors, combining %p-tau217 and Aβ42/40 did not provide further improvement, which could be due to the low number of participants who showed meaningful increases in Aβ burden during follow-up. Future studies in a larger sample and using highly precise approaches for quantitation of plasma p-tau217 and Aβ42/40 (such as for examples assays on fully automated platforms) are needed.

### Strengths and Limitations

The strengths of our study are the use of the state-of-the-art mass spectrometry methods for quantification of plasma biomarker levels,^42,43^ significant findings across 3 different thresholds (40 CL, 20 CL, 12 CL) to define elevated Aβ-PET, large sample size, and replication in 3 independent cohorts. There are also some limitations to consider. Participants in the BioFINDER and Knight ADRC cohorts are not fully representative of diverse populations. Thus, future investigations in cohorts with different ethnic, racial, and socioeconomic backgrounds are warranted. Plasma p-tau231 and GFAP levels were analyzed using immunoassays that in general might be somewhat less accurate than mass spectrometry–based methods. Finally, due to the lack of longitudinal plasma biomarker data, we were unable to determine if changes in plasma biomarker levels over time are better indicators of developing Aβ pathology.

## Conclusions

Results of this cohort study suggest that in CU individuals negative for Aβ, baseline plasma levels of both p-tau217 and Aβ42/40 were associated with cross-sectional and longitudinal measures of brain Aβ load. The utility of these biomarkers for identification of people at high risk of developing Aβ pathology (at individual level) for inclusion in primary AD prevention trials should be further explored in future studies.

## Notes

Supplement 1.
eMethods.
**eFigure 1.** Receiver Operating Characteristic (ROC) Curve Analysis for Predicting Abnormal Cerebrospinal Fluid (CSF) β-Amyloid (Aβ)42/40 Status in Cognitively Unimpaired Participants **eFigure 2.** Associations Between Plasma Biomarkers and β-Amyloid (Aβ) Positron Emission Tomography (Aβ-PET) at Baseline in Cognitively Unimpaired Participants With Subthreshold Baseline Aβ-PET in the Knight ADRC **eFigure 3.** Associations Between Plasma Biomarkers and Longitudinal Changes in β-Amyloid (Aβ) Positron Emission Tomography (Aβ-PET) in Cognitively Unimpaired Participants With Normal Baseline Aβ-PET in the Knight ADRC **eTable 1.** Demographic and Clinical Characteristics of CU Participants With Subthreshold Baseline Aβ-PET (<40 CL), BioFINDER-2 **eTable 2.** Associations of %p-Tau217 and Aβ42/40 With CSF Aβ42/40 and Aβ-PET, Cross-Sectional Analysis in BioFINDER-2 **eTable 3.** Associations of Plasma %p-Tau217, Aβ42/40, p-Tau231, and GFAP With CSF Aβ42/40 and Aβ-PET, Cross-Sectional Analysis in BioFINDER-2 **eTable 4.** Associations of Combinations of Plasma %p-Tau217, Aβ42/40, p-Tau231, and GFAP With CSF Aβ42/40 and Aβ-PET, Cross-Sectional Analysis in BioFINDER-2 **eTable 5.** Associations of Baseline Plasma Biomarkers (%p-Tau217, Aβ42/40, p-Tau231, and GFAP) With Baseline Aβ-PET in CU Participants With Subthreshold Baseline Aβ-PET (< 40 CL), BioFINDER-2 **eTable 6.** Associations Between Plasma Biomarkers and Aβ-PET From Table 3 Additionally Adjusting for Baseline Aβ-PET, BioFINDER-2 **eTable 7.** Associations of Baseline %p-Tau217 and Aβ42/40 With Longitudinal Aβ-PET in CU Participants With Subthreshold Baseline Aβ-PET, Linear Mixed-Effects Models, BioFINDER-2 **eTable 8.** Performance of %p-Tau217 and Aβ42/40 When Differentiating CU Participants With Subthreshold Baseline Aβ-PET (<40 CL, <20 CL or <12 CL) as Accumulators vs Nonaccumulators, BioFINDER-2 **eTable 9.** Performance of %p-Tau217 and Aβ42/40 When Identifying CU Participants With Subthreshold Baseline Aβ-PET (< 40 CL, <20 CL or <12 CL) Who Progressed to Aβ-PET Positivity, BioFINDER-2 ** eTable 10.** Demographic and Clinical Characteristics, Knight ADRC ** eTable 11.** Associations of %p-Tau217 and Aβ42/40 With CSF Aβ42/40 and Aβ-PET, Cross-Sectional Analysis in the Knight ADRC ** eTable 12.** Demographic and Clinical Characteristics of CU Participants With Subthreshold Baseline Aβ status, Knight ADRC, and BioFINDER-1 ** eTable 13.** Associations of Baseline %p-Tau217 and Aβ42/40 With Baseline and Longitudinal Aβ-PET in CU Participants With Subthreshold Aβ-PET (< 40 CL), Knight ADRC ** eTable 14.** Performance of %p-Tau217 and Aβ42/40 When Differentiating CU Participants With Subthreshold Baseline Aβ-PET (<40 CL) as Accumulators vs Nonaccumulators, Knight ADRC **eTable 15.** Associations of Baseline %p-tau217 and Aβ42/40 With Longitudinal CSF Aβ42/40 in CU Participants With Normal Baseline CSF Aβ42/40, BioFINDER-1
eReferences

Supplement 2.
Data Sharing Statement.
