# Supplementary material for: Plasma Phosphorylated Tau 217 and Aβ42/40 to Predict Early Brain Aβ Accumulation in People Without Cognitive Impairment
Source: JAMA Neurol. 2024 Jul 28;81(9):947–57. doi: 10.1001/jamaneurol.2024.2619 (PMC11284634; doi:10.1001/jamaneurol.2024.2619)
Supplement: Supplement 1. — eMethods. eFigure 1. Receiver Operating Characteristic (ROC) Curve Analysis for Predicting Abnormal Cerebrospinal Fluid (CSF) β-Amyloid (Aβ)42/40 Status in Cognitively Unimpaired Participants eFigure 2. Associations Between Plasma Biomarkers and β-Amyloid (Aβ) Positron Emission Tomography (Aβ-PET) at Baseline in Cognitively Unimpaired Participants With Subthreshold Baseline Aβ-PET in the Knight ADRC eFigure 3. Associations Between Plasma Biomarkers and Longitudinal Changes in β-Amyloid (Aβ) Positron Emission Tomography (Aβ-PET) in Cognitively Unimpaired Participants With Normal Baseline Aβ-PET in the Knight ADRC eTable 1. Demographic and Clinical Characteristics of CU Participants With Subthreshold Baseline Aβ-PET (<40 CL), BioFINDER-2 eTable 2. Associations of %p-Tau217 and Aβ42/40 With CSF Aβ42/40 and Aβ-PET, Cross-Sectional Analysis in BioFINDER-2 eTable 3. Associations of Plasma %p-Tau217, Aβ42/40, p-Tau231, and GFAP With CSF Aβ42/40 and Aβ-PET, Cross-Sectional Analysis in BioFINDER-2 eTable 4. Associations of Combinations of Plasma %p-Tau217, Aβ42/40, p-Tau231, and GFAP With CSF Aβ42/40 and Aβ-PET, Cross-Sectional Analysis in BioFINDER-2 eTable 5. Associations of Baseline Plasma Biomarkers (%p-Tau217, Aβ42/40, p-Tau231, and GFAP) With Baseline Aβ-PET in CU Participants With Subthreshold Baseline Aβ-PET (< 40 CL), BioFINDER-2 eTable 6. Associations Between Plasma Biomarkers and Aβ-PET From Table 3 Additionally Adjusting for Baseline Aβ-PET, BioFINDER-2 eTable 7. Associations of Baseline %p-Tau217 and Aβ42/40 With Longitudinal Aβ-PET in CU Participants With Subthreshold Baseline Aβ-PET, Linear Mixed-Effects Models, BioFINDER-2 eTable 8. Performance of %p-Tau217 and Aβ42/40 When Differentiating CU Participants With Subthreshold Baseline Aβ-PET (<40 CL, <20 CL or <12 CL) as Accumulators vs Nonaccumulators, BioFINDER-2 eTable 9. Performance of %p-Tau217 and Aβ42/40 When Identifying CU Participants With Subthreshold Baseline Aβ-PET (< 40 CL, <20 CL or <12 CL) Who Pro [file jamaneurol-e242619-s001.pdf]

Supplementary Online Content

Janelidze S, Barthélemy NR, Salvadó G, et al. Plasma phosphorylated tau 217 and Aβ42/40 to predict early brain Aβ accumulation in people without cognitive impairment. *JAMA Neurol*. Published online July 28, 2024. doi:10.1001/jamaneurol.2024.2619

eMethods.

eFigure 1. Receiver Operating Characteristic (ROC) Curve Analysis for Predicting Abnormal Cerebrospinal Fluid (CSF) β-Amyloid (Aβ)42/40 Status in Cognitively Unimpaired Participants

eFigure 2. Associations Between Plasma Biomarkers and β-Amyloid (Aβ) Positron Emission Tomography (Aβ-PET) at Baseline in Cognitively Unimpaired Participants With Subthreshold Baseline Aβ-PET in the Knight ADRC

eFigure 3. Associations Between Plasma Biomarkers and Longitudinal Changes in β-Amyloid (Aβ) Positron Emission Tomography (Aβ-PET) in Cognitively Unimpaired Participants With Normal Baseline Aβ-PET in the Knight ADRC

eTable 1. Demographic and Clinical Characteristics of CU Participants With Subthreshold Baseline Aβ-PET (<40 CL), BioFINDER-2

eTable 2. Associations of %p-Tau217 and Aβ42/40 With CSF Aβ42/40 and Aβ-PET, Cross-Sectional Analysis in BioFINDER-2

eTable 3. Associations of Plasma %p-Tau217, Aβ42/40, p-Tau231, and GFAP With CSF Aβ42/40 and Aβ-PET, Cross-Sectional Analysis in BioFINDER-2

eTable 4. Associations of Combinations of Plasma %p-Tau217, Aβ42/40, p-Tau231, and GFAP With CSF Aβ42/40 and Aβ-PET, Cross-Sectional Analysis in BioFINDER-2

eTable 5. Associations of Baseline Plasma Biomarkers (%p-Tau217, Aβ42/40, p-Tau231, and GFAP) With Baseline Aβ-PET in CU Participants With Subthreshold Baseline Aβ-PET (< 40 CL), BioFINDER-2

eTable 6. Associations Between Plasma Biomarkers and Aβ-PET From Table 3 Additionally Adjusting for Baseline Aβ-PET, BioFINDER-2

eTable 7. Associations of Baseline %p-Tau217 and Aβ42/40 With Longitudinal Aβ-PET in CU Participants With Subthreshold Baseline Aβ-PET, Linear Mixed-Effects Models, BioFINDER-2

eTable 8. Performance of %p-Tau217 and Aβ42/40 When Differentiating CU Participants With Subthreshold Baseline Aβ-PET (<40 CL, <20 CL or <12 CL) as Accumulators vs Nonaccumulators, BioFINDER-2

eTable 9. Performance of %p-Tau217 and Aβ42/40 When Identifying CU Participants With Subthreshold Baseline Aβ-PET (< 40 CL, <20 CL or <12 CL) Who Progressed to Aβ-PET Positivity, BioFINDER-2

eTable 10. Demographic and Clinical Characteristics, Knight ADRC

© 2024 Janelidze S et al. *JAMA Neurology*.

eTable 11. Associations of %p-Tau217 and Aβ42/40 With CSF Aβ42/40 and Aβ-PET, Cross-Sectional Analysis in the Knight ADRC

eTable 12. Demographic and Clinical Characteristics of CU Participants With Subthreshold Baseline Aβ status, Knight ADRC, and BioFINDER-1

eTable 13. Associations of Baseline %p-Tau217 and Aβ42/40 With Baseline and Longitudinal Aβ-PET in CU Participants With Subthreshold Aβ-PET (< 40 CL), Knight ADRC

eTable 14. Performance of %p-Tau217 and Aβ42/40 When Differentiating CU Participants With Subthreshold Baseline Aβ-PET (<40 CL) as Accumulators vs Nonaccumulators, Knight ADRC

eTable 15. Associations of Baseline %p-tau217 and Aβ42/40 With Longitudinal CSF Aβ42/40 in CU Participants With Normal Baseline CSF Aβ42/40, BioFINDER-1

eReferences

This supplementary material has been provided by the authors to give readers additional information about their work.

© 2024 Janelidze S et al. *JAMA Neurology*.

eMethods

Participants

The study comprised 495 CU participants from the Swedish BioFINDER-2 study (NCT03174938[1]). For validation we included CU individuals from two independent cohorts, the Knight ADRC at Washington University in St. Louis (N=283) and the Swedish BioFINDER-1 study (NCT01208675[2], N=205). All included participants had plasma p-tau217 (Knight ADRC BioFINDER-2: liquid chromatography-tandem mass spectrometry [LC-MS/MS]; BioFINDER-1: Lilly immuno-assay) and Aβ42/40 (LC-MS/MS) assessments.

In the BioFINDER cohorts, the CU group consisted of cognitively healthy controls (BioFINDER-2, n=360; BioFINDER-1, n=144) and participants with subjective cognitive decline (SCD; BioFINDER-2; n=135; BioFINDER-2; n=61) who performed within normal ranges on a large cognitive test battery (i.e., did not have MCI or dementia).[3] The neuropsychological battery covered the domains attention/executive function (Trail Making Test A and B and Symbol Digit Modalities Test), verbal ability (verbal fluency animals and the 15-word short version of the Boston Naming Test), memory (10-word delayed recall from the Alzheimer's Disease (AD) Assessment Scale [ADAS]), and visuospatial function (incomplete letters and cube analysis from the Visual Object and Space Perception battery [VOSP]). Patients with cognitive symptoms performing within normal ranges of cognitive tests were considered to have SCD. Study participants were recruited at the secondary care Memory Clinic at Skåne University Hospital in Malmö or the hospital of Ängelholm in Sweden, between 2017 and 2022 in BioFINDER-2 and between 2009 and 2015 in BioFINDER-1.

Participants in the Knight ADRC cohort at Washington University in St. Louis were community-dwelling volunteers enrolled in studies of memory and aging between 1988 and 2019. All participants underwent a comprehensive clinical assessment that included a detailed interview of a collateral source, a neurological examination of the participant, the Clinical Dementia Rating® (CDR®)[4], Clinical Dementia Rating Sum of Boxes (CDR-SB), and the Mini-Mental State Examination (MMSE).[5] Individuals with CDR score of 0.5 were classified as cognitively unimpaired.

Further details of inclusion/exclusion criteria for all 3 cohorts have been previously described[1, 6, 7]. The BioFINDER studies were approved by the Regional Ethics Committee in Lund, Sweden and all participants provided written informed consent. All procedures at Knight ADRC were approved by the Washington University Human Research Protection Office and written informed consent was obtained from each participant or their legally authorized representative when appropriate.

Plasma and CSF analysis

Plasma levels of Aβ42 and Aβ40 were analyzed using LC-MS/MS at the Department of Neurology, Washington University School of Medicine (BioFINDER) or at C_2N Diagnostics (Knight ADRC).[8, 9] In the BioFINDER-2 and Knight ADRC cohorts, plasma levels of phosphorylated and non-phosphorylated tau peptides were quantified using the multiplex LC-MS/MS developed at the Department of Neurology, Washington University School of Medicine. In BioFINDER-2, plasma Aβ and p-tau were analyzed with a multiplex assay using a single 1ml plasma aliquot. %p-tau217 was calculated as the ratio of tau phosphorylated at T217 residue divided by the concentration of the corresponding non-phosphorylated tau.[10, 11] In BioFINDER-1, plasma concentration of p-tau217 was determined at Lund University using an immuno-assay developed by Lilly Research Laboratories.[12, 13] Plasma levels of p-tau231 and GFAP were measured using in-house Simoa immuno-assay developed at the University of Gothenburg and commercially available Simoa Discovery immuno-assay (Quanterix), respectively.[14, 15]

CSF levels of Aβ40 and Aβ42 were assessed using Roche Elecsys immuno-assay and NeuroToolKit, respectively, on cobas e 411 and e 601 instruments (Roche Diagnostics). In the Knight ADRC cohort and for 10 BioFINDER-2 participants CSF Aβ40 and Aβ42 were measured with Lumipulse G (Fujirebio) immuno-assays. CSF Aβ status (negative/positive) was determined using the CSF Aβ42/40 ratio based on previously described cutoffs.[16-19] All samples were analyzed by staff blinded to the clinical data.

Aβ-PET image acquisition and processing

Aβ-PET imaging in BioFINDER-2 was performed on a digital GE Discovery MI scanner as previously described.[1] Scans were acquired 90-110 min after the injection of ~185 MBq [^{18}F]Flutemetamol. Standardized uptake value ratios (SUVR) were calculated using the whole cerebellum as the reference region. FreeSurfer (version 5.3) parcellation of the T1-weighted MRI scan was applied to the PET data transformed to participants' native T1 space to extract mean regional SUVR values for each participant in predefined neocortical regions of interest including prefrontal, lateral temporal, parietal, anterior cingulate, and posterior cingulate/precuneus.[20] BioFINDER-2 participants underwent their first Aβ-PET scan at baseline visit within 1 year of blood collection.

Participants at the Knight ADRC underwent Aβ-PET using either ^{18}F-AV45 (Florbetapir) or ^{11}C-Pittsburgh Compound B (PiB). Aβ-PET scans were performed in coordination with a 3 Tesla structural MRI scan.[6] T_1-weighted MRIs were processed using Freesurfer 5.3 to generate regions of interest used for the processing of PET data. Estimates of regional volumes were adjusted for intracranial volume using a regression approach. Data from the 30-60 minutes post-injection window for PiB and the 50-70 minutes window for Florbetapir were converted to SUVRs using the cerebellar grey as a reference. Values from the following regions were averaged together to represent mean cortical SUVR for Florbetapir

© 2024 Janelidze S et al. *JAMA Neurology*.

or PiB: bilateral orbitofrontal, medial orbitofrontal, rostral middle frontal, superior frontal, superior temporal, middle temporal, and precuneus. Aβ-PET scans performed with the same tracer within each individual were included in the longitudinal analysis and only if the first scan was performed not more than five years before blood collection.
SUVR values for ^{18}F-Flutemetamol, PiB and ^{18}F-AV45 were then transformed to Centiloids (CL)[21] for better comparability within this study, and with other studies. In the main analysis, similar to the A45 trial of the AHEAD 3-45 Study, [22] we used a cutoff of <40 CL to define subthreshold Aβ levels and identify CU individuals who did not have elevated brain Aβ at the visit closest to plasma collection. We also performed sensitivity analyses for 20 CL and 12 CL thresholds.[23, 24] To categorize study participants as Aβ accumulators/non-accumulators, we applied a previously defined threshold of >3.0 CL/year.[25]

© 2024 Janelidze S et al. *JAMA Neurology*.

eFigure 1

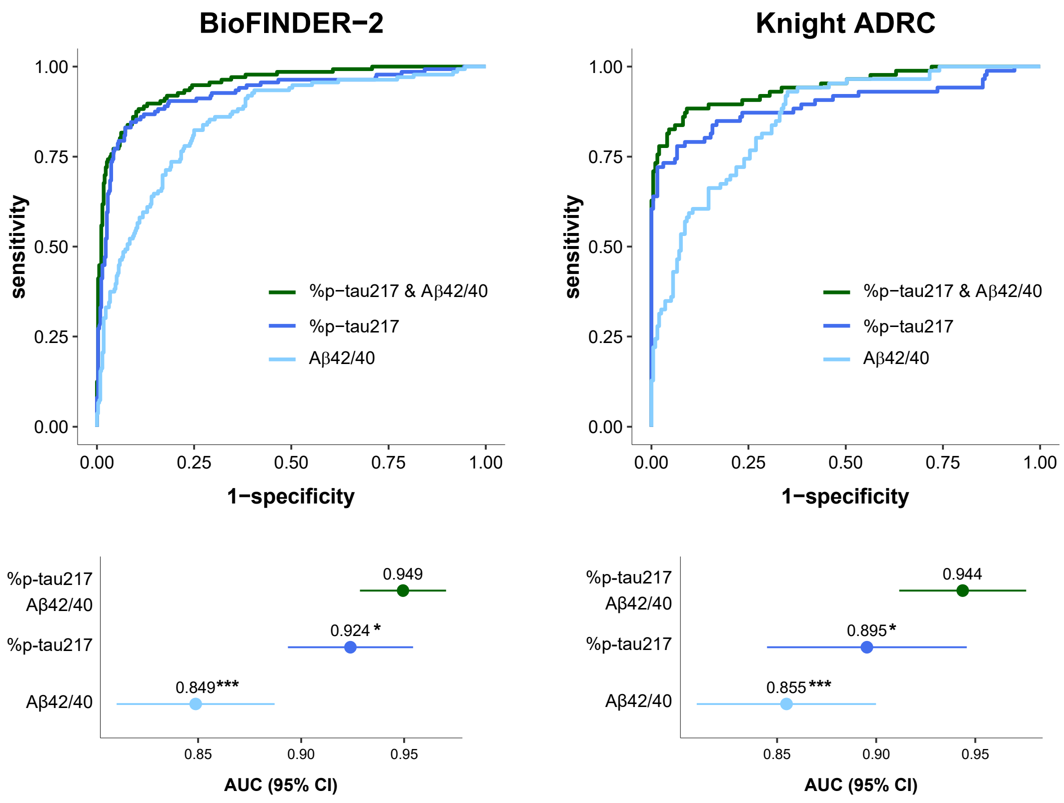

Receiver operating characteristic (ROC) curve analysis for predicting abnormal cerebrospinal fluid (CSF) β-amyloid (Aβ)42/40 status in cognitively unimpaired participants. ROC curve analysis for detecting abnormal CSF Aβ42/40 in cognitively unimpaired (CU) from the BioFINDER-2 (N=492) and Knight ADRC cohorts (N=283). Area under the curve (AUC) of two ROC curves were compared with the DeLong test.

© 2024 Janelidze S et al. *JAMA Neurology*.

eFigure 2

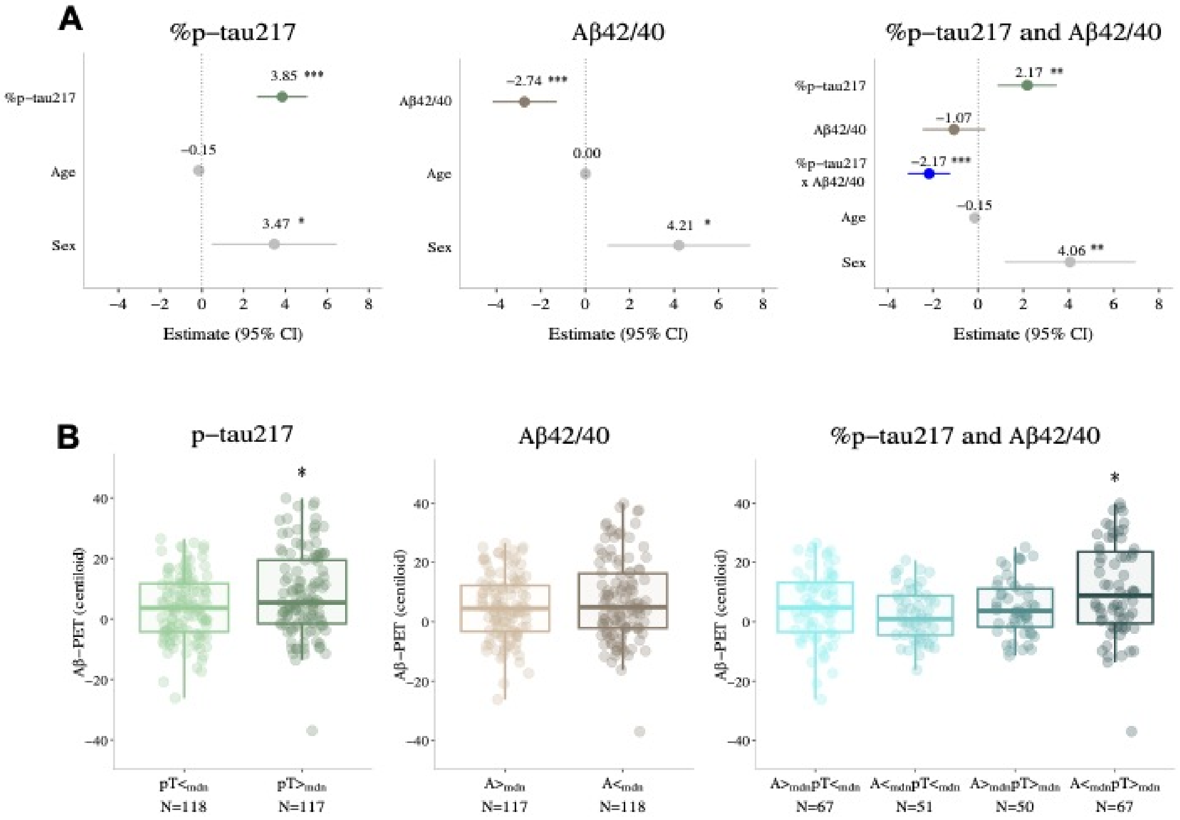

Associations between plasma biomarkers and β-amyloid (Aβ) positron emission tomography (Aβ-PET) at baseline in cognitively unimpaired participants with subthreshold baseline Aβ-PET in the Knight ADRC. (**A**) Estimates and 95%CI from linear regression models including continuous measures of %p-tau217, Aβ42/40 or %p-tau217, Aβ42/40 and their interaction as well as age and sex as predictors and Aβ-PET as outcome. Log-transformed and z-scored plasma biomarkers were used in all regression models. Aβ-PET status was defined by 40 CL threshold. (**B**) Differences in Aβ-PET centiloid values between participants with plasma biomarkers levels below or above median (mdn).

© 2024 Janelidze S et al. *JAMA Neurology*.

eFigure 3

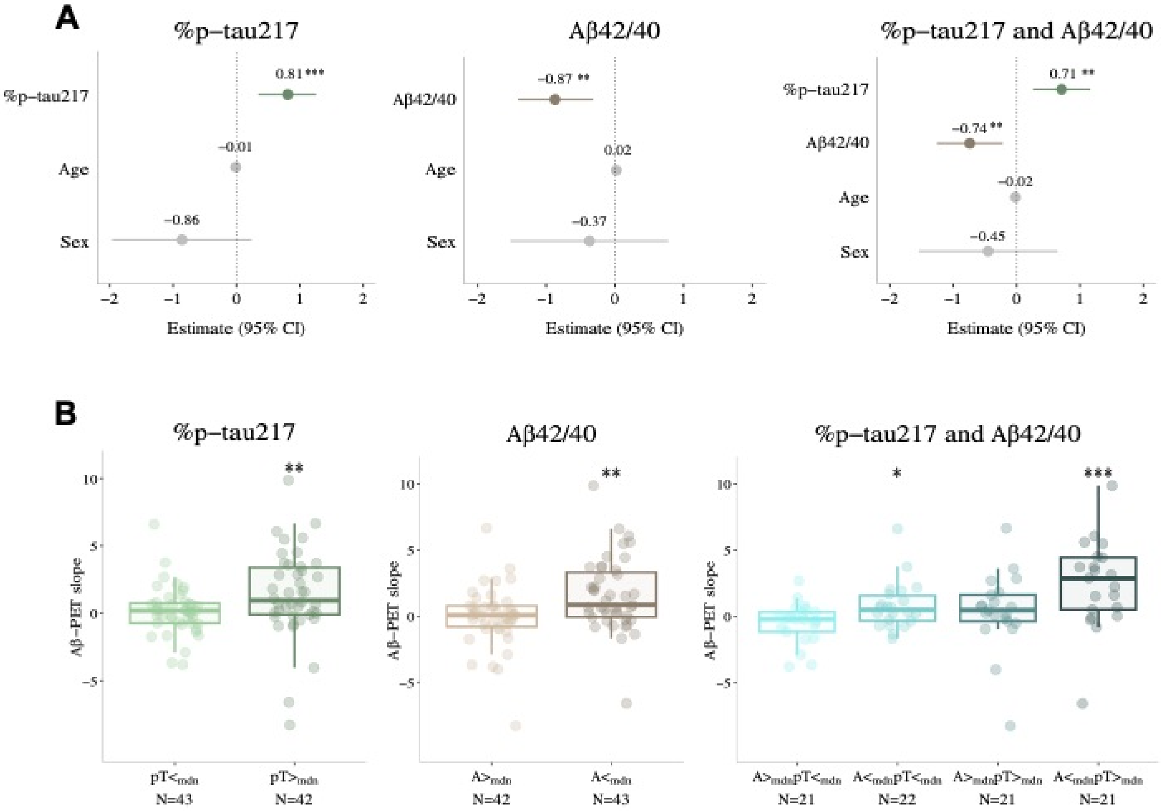

Associations between plasma biomarkers and longitudinal changes in β-amyloid (Aβ) positron emission tomography (Aβ-PET) in cognitively unimpaired participants with normal baseline Aβ-PET in the Knight ADRC. (**A**) Associations between baseline plasma biomarkers and longitudinal changes in Aβ-PET were tested using linear regression models including continuous measures of %p-tau217, Aβ42/40 or both biomarkers as well as age and sex as predictors and subject-specific slopes of Aβ-PET as outcome. Subject-specific slopes of Aβ-PET were derived using subject-level linear regression models with longitudinal Aβ-PET as outcome and time since plasma collection as predictor. Log-transformed and z-scored plasma biomarkers were used in all regression models. Aβ-PET status was defined by 40 CL threshold. (**B)** Differences in Aβ-PET centiloid values between participants with plasma biomarkers levels below or above median (mdn).

© 2024 Janelidze S et al. *JAMA Neurology*.

eTable 1. Demographic and clinical characteristics of CU participants with subthreshold baseline Aβ-PET (<40 CL), BioFINDER-2

	Cognitively unimpaired Aβ-PET <40 CL at baseline
N	224
Age, years	62.6 (53.9 - 74.2)
Male, n (%)	109 (48.7%)
MMSE	29.0 (28.0 - 30.0)
Education, years	13.0 (11.0 - 15.0)
APOE ε4 positivity, n (%)	86 (38.4%)
CSFAβ42/40 positivity, n (%)	34 (15.2%)
No. of participants with 2/3 PET visits	144/80
Plasma %p-tau217	0.701 (0.595 - 0.873)
Plasma Aβ42/40	0.121 (0.114 - 0.127)
Plasma p-tau231, pg/ml	4.8 (3.7-6.1)
Plasma GFAP, pg/ml	95.6 (65.4-138.8)

Data are shown as median (interquartile range) unless otherwise specified.
Education and plasma p-tau231 data were missing for 2 and 1 participants, respectively.
Abbreviations: Aβ, amyloid-β; CSF, cerebrospinal fluid; GFAP, glial fibrillary acidic protein; MMSE, Mini Mental State Examination; PET, positron emission tomography; p-tau, phosphorylated tau.

© 2024 Janelidze S et al. *JAMA Neurology*.

eTable 2. Associations of %p-tau217 and Aβ42/40 with CSF Aβ42/40 and Aβ-PET, cross-sectional analysis in BioFINDER-2

	AUC (95% CI)	*P-value* [a]
**CSF Aβ42/40** [b]	**136 Aβ+, 356 Aβ-**	
%p-tau217	0.924 (0.894-0.954)	0.024
Aβ42/40	0.849 (0.810-0.887)	2.2e-09
%p-tau217 & Aβ42/40	0.949 (0.929-0.970)	NA
**Aβ-PET 40 CL**	**65 A+, 384 A-**	
%p-tau217	0.976 (0.963-0.989)	0.71
Aβ42/40	0.769 (0.705-0.834)	<0.001
%p-tau217 & Aβ42/40	0.976 (0.963-0.989)	1.1e-10
**Aβ-PET 20 CL**	**93 Aβ+, 356 βA-**	
%p-tau217	0.969 (0.953-0.984)	0.91
Aβ42/40	0.801 (0.751-0.852)	2.8e-12
%p-tau217 & Aβ42/40	0.968 (0.952-0.984)	NA
**Aβ-PET 12 CL**	**102 Aβ+, 347 Aβ-**	
%p-tau217	0.974 (0.960-0.987)	0.99
Aβ42/40	0.817 (0.770-0.864)	2.6e-12
%p-tau217 & Aβ42/40	0.974 (0.960-0.988)	NA

Data are from ROC curve analysis with CSF Aβ42/40 or Aβ-PET status as outcome.
[a] AUC of the model combining %p-tau217 & Aβ42/40 were compared to AUCs of %p-tau217 and Aβ42/40 using the DeLong test.
[b] CSF Aβ42/40 status was defined using thresholds described in the Method.
Abbreviations: Aβ, amyloid-β; AUC, area under the curve; CI, confidence interval; CL, centiloid; CSF, cerebrospinal fluid; NA, not applicable; ROC, Receiver Operating Characteristic; PET, positron emission tomography; p-tau, phosphorylated tau.

© 2024 Janelidze S et al. *JAMA Neurology*.

eTable 3. Associations of plasma %p-tau217, Aβ42/40, p-tau231 and GFAP with CSF Aβ42/40 and Aβ-PET, cross-sectional analysis in BioFINDER-2

	AUC (95% CI)	*P-value* [a]
**CSF Aβ42/40** [b]	***99 Aβ+, 314 Aβ-***	
%p-tau217	0.931 (0.897-0.965)	NA
Aβ42/40	0.843 (0.795-0.89)	0.003
p-tau231	0.874 (0.834-0.914)	0.009
GFAP	0.762 (0.715-0.81)	2.0e-11
**Aβ-PET 40 CL**	***49 Aβ+, 330 Aβ-***	
%p-tau217	0.984 (0.972-0.995)	NA
Aβ42/40	0.751 (0.671-0.832)	5.8e-09
p-tau231	0.890 (0.847-0.933)	2.0e-05
GFAP	0.832 (0.781-0.884)	7.7e-09
**Aβ-PET 20 CL**	***69 Aβ+, 310 Aβ-***	
%p-tau217	0.977 (0.963-0.99)	NA
Aβ42/40	0.797 (0.735-0.858)	1.3e-08
p-tau231	0.857 (0.811-0.904)	1.1e-07
GFAP	0.811 (0.762-0.86)	1.1e-11
**Aβ-PET 12 CL**	***78 Aβ+, 301 Aβ-***	
%p-tau217	0.980 (0.968-0.992)	NA
Aβ42/40	0.816 (0.76-0.872)	1.7-e08
p-tau231	0.876 (0.834-0.917)	4.5e-07
GFAP	0.801 (0.754-0.849)	6.2e-14

Results are from ROC curve analysis with CSF Aβ42/40 or Aβ-PET status as outcome in the dataset that included all plasma biomarkers.
[a] AUCs of the models combining plasma biomarkers were compared to AUC of %p-tau217 using the DeLong test.
[b] CSF Aβ42/40 status was defined using thresholds described in the Method.
Abbreviations: Aβ, amyloid-β; AUC, area under the curve; CI, confidence interval; CL, centiloid; CSF, cerebrospinal fluid; GFAP, glial fibrillary acidic protein; NA, not applicable; ROC, Receiver Operating Characteristic; PET, positron emission tomography; p-tau, phosphorylated tau.

© 2024 Janelidze S et al. *JAMA Neurology*.

eTable 4. Associations of combinations of plasma %p-tau217, Aβ42/40, p-tau231 and GFAP with CSF Aβ42/40 and Aβ-PET, cross-sectional analysis in BioFINDER-2

	AUC (95% CI)	*P-value* [a]
**CSF Aβ42/40** [b]	***99 Aβ+, 314 Aβ-***	
%p-tau217 & Aβ42/40	0.952 (0.927-0.977)	NA
p-tau231& GFAP	0.879 (0.841-0.917)	0.001
%p-tau217 & Aβ42/40 & p-tau231	0.970 (0.955-0.985)	0.07
%p-tau217 & Aβ42/40 & GFAP	0.953 (0.929-0.977)	0.57
**Aβ-PET 40 CL**	***49 Aβ+, 330 Aβ-***	
%p-tau217 & Aβ42/40	0.984 (0.973-0.995)	NA
p-tau231& GFAP	0.896 (0.855-0.936)	1.9e-05
%p-tau217 & Aβ42/40 &	0.984 (0.974-0.995)	0.70
%p-tau217 & Aβ42/40 & GFAP	0.984 (0.973-0.995)	0.74
**Aβ-PET 20 CL**	***69 Aβ+, 310 Aβ-***	
%p-tau217 & Aβ42/40	0.975 (0.96-0.99)	NA
p-tau231& GFAP p-tau231	0.863 (0.82-0.906)	2.0e-07
%p-tau217 & Aβ42/40 &	0.977 (0.964-0.99)	0.51
%p-tau217 & Aβ42/40 & GFAP	0.976 (0.962-0.99)	0.65
**Aβ-PET 12 CL**	***78 Aβ+, 301 Aβ-***	
%p-tau217 & Aβ42/40	0.979 (0.965-0.993)	NA
p-tau231& GFAP p-tau231	0.876 (0.837-0.915)	4.3e-07
%p-tau217 & Aβ42/40 &	0.982 (0.972-0.993)	0.50
%p-tau217 & Aβ42/40 & GFAP	0.979 (0.965-0.992)	0.79

Results are from ROC curve analysis with CSF Aβ42/40 or Aβ-PET status as outcome in the dataset that included all plasma biomarkers.

[a] AUCs of the models combining plasma biomarkers were compared to AUCs of %p-tau217 and Aβ42/40 using the DeLong test.

[b] CSF Aβ42/40 status was defined using thresholds described in the Method.

Abbreviations: Aβ, amyloid-β; AUC, area under the curve; CI, confidence interval; CL, centiloid; CSF, cerebrospinal fluid; GFAP, glial fibrillary acidic protein; NA, not applicable; ROC, Receiver Operating Characteristic; PET, positron emission tomography; p-tau, phosphorylated tau.

© 2024 Janelidze S et al. *JAMA Neurology*.

eTable 5. Associations of baseline plasma biomarkers (%p-tau217, Aβ42/40, p-tau231 and GFAP) with baseline Aβ-PET in CU participants with subthreshold baseline Aβ-PET (< 40 CL), BioFINDER-2

	**Model**			**%P-tau217**	**Aβ42/40**	**P-tau231**
	**N**	Adjusted R^2	AIC	β (95% CI), p-value	β (95% CI), p-value	β (95% CI), p-value
%P-tau217 & Aβ42/40	330	0.400	2388.9	4.24 (3.34, 5.13), 1.8e-18	-2.50 (-3.42, -1.58), 1.7e-07	NA
%P-tau217 & Aβ42/40 & p-tau231	330	0.403	2388.4	3.98 (3.03, 4.93), 4.1e-15	-2.43 (-3.36, -1.51), 3.7e-07	0.73 (-0.19, 1.66), 0.12
%P-tau217 & Aβ42/40	338	0.390	2454.0	4.19 (3.29, 5.09), 5.9e-18	-2.61 (-3.52, -1.70), 3.6e-08	NA
%P-tau217 & Aβ42/40 & GFAP	338	0.389	2455.5	4.09 (3.14, 5.03), 6.8e-16	-2.65 (-3.56, -1.73), 2.9e-08	0.47 (-0.82, 1.76), 0.47

Data are from linear regression models including continuous measures of %p-tau217 and Aβ42/40 or %p-tau217, Aβ42/40 together with either p-tau231 or GFAP as predictors and baseline Aβ-PET as outcome. The models also included age and sex as covariates. Abbreviations: AIC, akaike information criterion; Aβ, amyloid-β; CI, confidence interval; CL, centiloid; CU, cognitively unimpaired; GFAP, glial fibrillary acidic protein; NA, not applicable; PET, positron emission tomography; p-tau, phosphorylated tau.

© 2024 Janelidze S et al. *JAMA Neurology*.

eTable 6. Associations between plasma biomarkers and Aβ-PET from Table 3 additionally adjusting for baseline Aβ-PET, BioFINDER-2

	Model		%p-tau217	Aβ42/40	%p-tau217 x Aβ42/40	Baseline Aβ-PET
	Adjusted R^2	AIC	β (95% CI), p-value	β (95% CI), p-value	β (95% CI), p-value	β (95% CI), p-value
**< 40 CL (N=224)**						
model 1, %p-tau217	0.542	753.3	0.55 (0.37, 0.74), 1.9e-08	NA	NA	0.09 (0.07, 0.11), 3.3e-17
model 2, Aβ42/40	0.497	774.3	NA	-0.30 (-0.48, -0.13), 0.0009	NA	0.10 (0.09, 0.12), 1.9e-23
model 3, %p-tau217 & Aβ42/40	0.567	742.8	0.47 (0.28, 0.66), 1.5e-06	-0.22 (-0.39, -0.05), 0.011	-0.14 (-0.27, -0.02), 0.027	0.07 (0.05, 0.09), 4.3e-10
model 4, baseline Aβ-PET	0.473	783.6	NA	NA	NA	0.12 (0.10, 0.13), 2.6e-30
**< 20 CL (N=209)**						
model 1, %p-tau217	0.260	701.0	0.56 (0.37, 0.75), 3.4e-08	NA	NA	0.06 (0.03, 0.09), 4.8e-05
model 2, Aβ42/40	0.178	723.2	NA	-0.28 (-0.47, -0.10), 0.003	NA	0.07 (0.04, 0.10), 1.4e-06
model 3, %p-tau217 & Aβ42/40	0.333	681.2	0.47 (0.29, 0.66), 1.2e-06	-0.22 (-0.39, -0.06), 0.009	-0.36 (-0.54, -0.18), 0.0001	0.04 (0.01, 0.07), 0.003
model 4, baseline Aβ-PET	0.145	730.3	NA	NA	NA	0.08 (0.05, 0.11), 1.1e-07
**< 12 CL (N=203)**						
model 1, %p-tau217	0.132	648.0	0.47 (0.29, 0.65), 7.8e-07	NA	NA	0.01 (-0.02, 0.04), 0.6
model 2, Aβ42/40	0.047	667.0	NA	-0.21 (-0.38, -0.04), 0.015	NA	0.01 (-0.02, 0.05), 0.35
model 3, %p-tau217 & Aβ42/40	0.172	640.5	0.43 (0.25, 0.61), 3.6e-06	-0.19 (-0.35, -0.03), 0.021	-0.23 (-0.41, -0.04), 0.017	0.01 (-0.02, 0.04), 0.66
model 4, baseline Aβ-PET	0.023	671.1	NA	NA	NA	0.02 (-0.01, 0.05), 0.27

Data are from linear regression models including continuous measures of %p-tau217 (model 1), Aβ42/40 (model 2), %p-tau217, Aβ42/40 and their interaction (model 3) or none of the plasma biomarkers (model 4) to predict the subject-specific slopes of Aβ-PET. All models included age, sex, and baseline Aβ-PET as covariates. Subject-specific slopes of Aβ-PET were derived from linear mixed effects models including longitudinal Aβ-PET as outcome and time (years since baseline) as predictor.
Abbreviations: AIC, akaike information criterion; Aβ, amyloid-β; CI, confidence interval; CL, centiloid; CU, cognitively unimpaired; NA, not applicable; PET, positron emission tomography; p-tau, phosphorylated tau.

© 2024 Janelidze S et al. *JAMA Neurology*.

eTable 7. Associations of baseline %p-tau217 and Aβ42/40 with longitudinal Aβ-PET in CU participants with subthreshold baseline Aβ-PET, linear mixed effects models, BioFINDER-2

	%p-tau217	Aβ42/40	%p-tau217 * Aβ42/40
	β (95% CI), p-value	β (95% CI), p-value	β (95% CI), p-value
**< 40 CL (N=224)**			
model 1, %p-tau217	1.26 (1.04, 1.48), 2.1e-22	NA	NA
model 2, Aβ42/40	NA	-0.91 (-1.17, -0.64), 1.0e-10	NA
model 3, %p-tau217 & Aβ42/40	0.90 (0.66, 1.14), 1.0e-11	-0.44 (-0.67, -0.20), 0.0004	-0.29 (-0.45, -0.13), 0.0004
**< 20 CL (N=209)**			
model 1, %p-tau217	0.90 (0.66, 1.13), 3.9e-12	NA	NA
model 2, Aβ42/40	NA	-0.52 (-0.78, -0.27), 8.2e-05	NA
model 3, %p-tau217 & Aβ42/40	0.72 (0.49, 0.95), 5.0e-09	-0.36 (-0.58, -0.14), 0.002	-0.49 (-0.72, -0.26), 5.0e-05
**< 12 CL (N=203)**			
model 1, %p-tau217	0.68 (0.45, 0.91), 1.6e-08	NA	NA
model 2, Aβ42/40	NA	-0.33 (-0.57, -0.09), 0.007	NA
model 3, %p-tau217 & Aβ42/40	0.62 (0.40, 0.84), 1.1e-07	-0.28 (-0.49, -0.06), 0.012	-0.30 (-0.54, -0.06), 0.015

Estimates (β), 95% confidence interval (CI) and p-values are from linear mixed models with Aβ-PET as outcome variable and biomarker interaction with time as predictors (model 1: time*%p-tau217; model 2: time*Aβ42/40; model 3: time*%p-tau217*Aβ42/40). All models included continuous measures of plasma %p-tau217 and/or Aβ42/40 and age and sex as covariates.
Abbreviations: Aβ, amyloid-β; CI, confidence interval; CL, centiloid; CU, cognitively unimpaired; NA, not applicable; PET, positron emission tomography; p-tau, phosphorylated tau.

© 2024 Janelidze S et al. *JAMA Neurology*.

eTable 8. Performance of %p-tau217 and Aβ42/40 when differentiating CU participants with subthreshold baseline Aβ-PET (<40 CL, <20 CL or <12 CL) as accumulators vs non-accumulators, BioFINDER-2

	AUC (95% CI)	*P-value* [a]	*SN*	*SP*
**< 40 CL**	***32 accumulators, 192 non-accumulators***			
%p-tau217	0.949 (0.918-0.979)	0.48	0.84	0.90
Aβ42/40	0.821 (0.742-0.90)	1.6e-06	0.50	0.90
%p-tau217 & Aβ42/40	0.937 (0.886-0.987)	NA	0.84	0.90
**< 20 CL**	***17 accumulators, 192 non-accumulators***			
%p-tau217	0.931 (0.888-0.975)	0.18	0.76	0.90
Aβ42/40	0.749 (0.623-0.874)	6.0e-06	0.41	0.90
%p-tau217 & Aβ42/40	0.894 (0.806-0.982)	NA	0.71	0.90
**< 12 CL**	***11 accumulators, 192 non-accumulators***			
%p-tau217	0.912 (0.853-0.971)	0.10	0.64	0.90
Aβ42/40	0.653 (0.488-0.819)	3.3e-06	0.27	0.90
%p-tau217 & Aβ42/40	0.853 (0.733-0.972)	NA	0.55	0.90

Results are from ROC curve analysis with Aβ-PET accumulators / non-accumulators as outcome. Study participants were categorized as accumulators/non-accumulators using previously defined threshold of >3.0 CL/year.[25]
[a] AUCs of the models combining plasma biomarkers were compared to AUCs of %p-tau217 and Aβ42/40 using the DeLong test.
Abbreviations: Aβ, amyloid-β; AUC, area under the curve; CI, confidence interval; CL, centiloid; NA, not applicable; ROC, Receiver Operating Characteristic; PET, positron emission tomography; p-tau, phosphorylated tau.

© 2024 Janelidze S et al. *JAMA Neurology*.

eTable 9. Performance of %p-tau217 and Aβ42/40 when identifying CU participants with subthreshold baseline Aβ-PET (< 40 CL, <20 CL or <12 CL) who progressed to Aβ-PET positivity, BioFINDER-2

	AUC (95% CI)	*P-value [a]*	*SN*	*SP*
**< 40 CL**	***12 progressors, 212 non-progressors***			
%p-tau217	0.954 (0.912-0.997)	0.25	0.83	0.90
Aβ42/40	0.897 (0.82-0.973)	0.027	0.67	0.90
%p-tau217 & Aβ42/40	0.975 (0.953-0.997)	NA	1.00	0.90
**< 20 CL**	***10 progressors, 199 non-progressors***			
%p-tau217	0.917 (0.85-0.984)	0.34	0.80	0.90
Aβ42/40	0.765 (0.571-0.96)	0.002	0.50	0.90
%p-tau217 & Aβ42/40	0.868 (0.71-1.00)	NA	0.80	0.90
**< 12 CL**	***9 progressors, 194 non-progressors***			
%p-tau217	0.871 (0.78-0.963)	0.095	0.56	0.90
Aβ42/40	0.608 (0.383-0.832)	2.3e-04	0.33	0.90
%p-tau217 & Aβ42/40	0.793 (0.621-0.964)	NA	0.44	0.90

Results are from ROC curve analysis with Aβ-PET progressors / non-progressors as outcome.
[a] AUCs of the models combining plasma biomarkers were compared to AUCs of %p-tau217 and Aβ42/40 using the DeLong test.
Abbreviations: Aβ, amyloid-β; AUC, area under the curve; CI, confidence interval; CL, centiloid; NA, not applicable; ROC, Receiver Operating Characteristic; PET, positron emission tomography; p-tau, phosphorylated tau.

eTable 10. Demographic and clinical characteristics, Knight ADRC

	**Cognitively unimpaired**
N	283
Age, years	69.1 (63.3 - 74.3)
Male, n (%)	132 (46.6%)
MMSE	30.0 (29.0 - 30.0)
Education, years	16.0 (16.0 - 18.0)
Race (Black/White/Other), n	255/24/4
APOE ε4 positivity, n (%)	101 (35.7 %)
CSFAβ42/40 positivity, n (%)	86 (30.4%)
Plasma %p-tau217	0.646 (0.468 - 1.032)
Plasma Aβ42/40	0.101 (0.095 - 0.106)

Data are shown as median (interquartile range) unless otherwise specified.
Abbreviations: Aβ, amyloid-β; CSF, cerebrospinal fluid; MMSE, Mini Mental State Examination; PET, positron emission tomography; p-tau, phosphorylated tau

© 2024 Janelidze S et al. *JAMA Neurology*.

eTable 11. Associations of %p-tau217 and Aβ42/40 with CSF Aβ42/40 and Aβ-PET, cross-sectional analysis in the Knight ADRC

	AUC (95% CI)	*P-value [a]*
**CSF Aβ42/40 [b]**	***86 Aβ+, 197 Aβ-***	
%p-tau217	0.895 (0.845-0.946)	0.012
Aβ42/40	0.855 (0.810-0.900)	<0.001
%p-tau217 & Aβ42/40	0.944 (0.912-0.976)	NA
**Aβ-PET 40 CL**	***48 Aβ+, 235 Aβ-***	
%p-tau217	0.948 (0.918-0.977)	0.74
Aβ42/40	0.820 (0.762-0.877)	<0.001
%p-tau217 & Aβ42/40	0.951 (0.926-0.976)	NA
**Aβ-PET 20 CL**	***85 Aβ+, 198 Aβ-***	
%p-tau217	0.881 (0.83-0.933)	0.74
Aβ42/40	0.768 (0.705-0.831)	<0.001
%p-tau217 & Aβ42/40	0.878 (0.822-0.934)	NA
**Aβ-PET 12 CL**	***115 Aβ+, 168 Aβ-***	
%p-tau217	0.780 (0.718-0.841)	0.39
Aβ42/40	0.706 (0.643-0.768)	0.001
%p-tau217 & Aβ42/40	0.789 (0.728-0.850)	NA

Data are from ROC curve analysis with CSF Aβ42/40 or Aβ-PET status as outcome.
[a] AUC of the model combining %p-tau217 & Aβ42/40 were compared to AUCs of %p-tau217 and Aβ42/40 using the DeLong test.
[b] CSF Aβ42/40 status was defined using thresholds described in the Method.
Abbreviations: Aβ, amyloid-β; AUC, area under the curve; CI, confidence interval; CSF, cerebrospinal fluid; NA, not applicable; ROC, Receiver Operating Characteristic; p-tau, phosphorylated tau.

© 2024 Janelidze S et al. *JAMA Neurology*.

eTable 12. Demographic and clinical characteristics of CU participants with subthreshold baseline Aβ status, Knight ADRC and BioFINDER-1

	Knight ADRC Cognitively unimpaired Aβ-PET <40 CL at baseline	BioFINDER-1 Cognitively unimpaired CSF Aβ42/40 negative at baseline [a]
N	235	205
Age, years	68.3 (62.8 - 73.8)	72.4 (68.3 - 75.9)
Male, n (%)	114 (48.511%)	78 (38.0%)
MMSE	30.0 (29.0 - 30.0)	29.0 (28.0 - 30.0)
Education, years	16.0 (16.0 - 18.0)	12.0 (10.0 - 14.0)
Race (Black/White/Other), n	209/22/4	NA
APOE ε4 positivity, n (%)	70 (29.8%)	43 (21.4%)
CSFAβ42/40 positivity, n (%)	39 (16.6%)	0 (0%)
No. of participants with 2/3/4 PET visits	73/11/1	NA
No. of participants with 2/3/4/5 CSF visits	NA	67/67/66/6
Plasma %p-tau217	0.586 (0.450 - 0.754)	NA
Plasma p-tau217, pg/ml	NA	0.161 (0.133 - 0.200)
Plasma Aβ42/40	0.102 (0.097 - 0.108)	0.141 (0.135 - 0.147)

Data are shown as median (interquartile range) unless otherwise specified.
[a] CSF Aβ42/40 status was defined using CSF Aβ42/40 thresholds described in the Method.
APOE ε4 was missing in 4 participants in BioFINDER-1.
Abbreviations: Aβ, amyloid-β; CSF, cerebrospinal fluid; MMSE, Mini Mental State Examination; NA, not available or not applicable; PET, positron emission tomography; p-tau, phosphorylated tau.

© 2024 Janelidze S et al. *JAMA Neurology*.

eTable 13. Associations of baseline %p-tau217 and Aβ42/40 with baseline and longitudinal Aβ-PET in CU participants with subthreshold Aβ-PET (< 40 CL), Knight ADRC

	Model		%p-tau217	Aβ42/40	%p-tau217 * Aβ42/40
	Adjusted R^2	AIC	β (95% CI), p-value	β (95% CI), p-value	β (95% CI), p-value
**Baseline Aβ-PET (N=235)**					
model 1, %p-tau217	0.148	1821.3	3.85 (2.64, 5.05), 1.5e-09	NA	NA
model 2, Aβ42/40	0.058	1844.8	NA	-2.74 (-4.18, -1.29), 0.0002	NA
model 3, %p-tau217 & Aβ42/40	0.234	1798.1	2.17 (0.861, 3.47), 0.001	-1.07 (-2.45, 0.306), 0.13	-2.17 (-3.11, -1.23), 9.2e-06
**Longitudinal Aβ-PET (N=85)**					
model 1, %p-tau217	0.136	402.3	0.805 (0.345, 1.26), 0.0008	NA	NA
model 2, Aβ42/40	0.117	404.2	NA	-0.865 (-1.41, -0.323), 0.002	NA
model 3, %p-tau217 & Aβ42/40 [a]	0.206	396.2	0.710 (0.261, 1.16), 0.002	-0.742 (-1.26, -0.222), 0.006	NA

Data are from linear regression models including continuous measures of %p-tau217 (model 1), Aβ42/40 (model 2) or %p-tau217, Aβ42/40 and their interaction (model 3) as predictors and baseline Aβ-PET or subject-specific slopes of Aβ-PET as outcome. The models included age and sex, as covariates. Subject-specific slopes of Aβ-PET were derived from subject-specific linear regression models (one model fit for each subject) including longitudinal Aβ-PET as outcome and time (years since plasma collection) as predictor.
[a] Interaction between %p-tau217 and Aβ42/40 was not a significant predictor of Aβ-PET slopes and was not included in the model.
Abbreviations: Aβ, amyloid-β; AIC, akaike information criterion; CI, confidence interval; CL, centiloid; CU, cognitively unimpaired; NA, not applicable; PET, positron emission tomography; p-tau, phosphorylated tau.

eTable 14. Performance of %p-tau217 and Aβ42/40 when differentiating CU participants with subthreshold baseline Aβ-PET (<40 CL) as accumulators vs non-accumulators, Knight ADRC

	AUC (95% CI)	*P-value [a]*	*SN*	*SP*
**Aβ-PET CL 40**	***14 accumulators, 71 non-accumulators***			
%p-tau217	0.844 (0.688-1.00)	0.53	0.79	0.90
Aβ42/40	0.786 (0.634-0.937)	0.21	0.50	0.90
%p-tau217 & Aβ42/40	0.874 (0.756-0.993)	NA	0.79	0.90

Results are from ROC curve analysis with Aβ-PET accumulators / non-accumulators as outcome. Study participants were categorized as accumulators/non-accumulators using previously defined threshold of >3.0 CL/year.[25]
[a] AUCs of the models combining plasma biomarkers were compared to AUCs of %p-tau217 and Aβ42/40 using the DeLong test.
Abbreviations: Aβ, amyloid-β; AUC, area under the curve; CI, confidence interval; CL, centiloid; NA, not applicable; ROC, Receiver Operating Characteristic; PET, positron emission tomography; p-tau, phosphorylated tau.

© 2024 Janelidze S et al. *JAMA Neurology*.

eTable 15. Associations of baseline %p-tau217 and Aβ42/40 with longitudinal CSF Aβ42/40 in CU participants with normal baseline CSF Aβ42/40, BioFINDER-1

	**Model**		**P-tau217 Lilly**	**Aβ42/40**
	Adjusted R^2	AIC	β (SE), p-value	β (SE), p-value
model 1, p-tau217 Lilly	0.038	-2079.2	-0.00030 (-0.0005, -0.0001), 0.0035	NA
model 2, Aβ42/40	0.085	-2089.5	NA	0.00048 (0.0003, 0.0007), 1.6e-05
model 3, p-tau217 Lilly & Aβ42/40	0.109	-2094.0	-0.00025 (-0.0004, -0.00005), 0.012	0.00044 (0.0002, 0.0006), 5.3e-05

Data are from linear regression models including continuous measures of %p-tau217 (model 1), Aβ42/40 (model 2) or both (model 3) to predict the subject-specific slopes of CSF Aβ42/40. The models included age and sex as covariates. Subject-specific slopes of CSF Aβ42/40 were derived from linear mixed effects models including longitudinal CSF Aβ42/40 as outcome and time (years since baseline) as predictor.
Abbreviations: Aβ, amyloid-β; AIC, akaike information criterion; CI, confidence interval; CSF, cerebrospinal fluid; CU, cognitively unimpaired; NA, not applicable; p-tau, phosphorylated tau.

© 2024 Janelidze S et al. *JAMA Neurology*.

eReferences

1. Palmqvist S, Janelidze S, Quiroz YT, et al. Discriminative Accuracy of Plasma Phospho-tau217 for Alzheimer Disease vs Other Neurodegenerative Disorders. *JAMA*. 2020;doi:10.1001/jama.2020.12134
2. Palmqvist S, Janelidze S, Stomrud E, et al. Performance of Fully Automated Plasma Assays as Screening Tests for Alzheimer Disease-Related beta-Amyloid Status. *JAMA Neurol*. 2019;doi:10.1001/jamaneurol.2019.1632
3. Jack CR, Jr., Bennett DA, Blennow K, et al. NIA-AA Research Framework: Toward a biological definition of Alzheimer's disease. *Alzheimers Dement*. 2018;14(4):535-562. doi:10.1016/j.jalz.2018.02.018
4. Morris JC. The Clinical Dementia Rating (CDR): current version and scoring rules. *Neurology*. 1993;43(11):2412-4. doi:10.1212/wnl.43.11.2412-a
5. Folstein MF, Folstein SE, McHugh PR. "Mini-mental state". A practical method for grading the cognitive state of patients for the clinician. *J Psychiatr Res*. 1975;12(3):189-98. doi:10.1016/0022-3956(75)90026-6
6. Barthelemy NR, Saef B, Li Y, et al. CSF tau phosphorylation occupancies at T217 and T205 represent improved biomarkers of amyloid and tau pathology in Alzheimer's disease. *Nat Aging*. 2023;3(4):391-401. doi:10.1038/s43587-023-00380-7
7. Palmqvist S, Zetterberg H, Blennow K, et al. Accuracy of brain amyloid detection in clinical practice using cerebrospinal fluid beta-amyloid 42: a cross-validation study against amyloid positron emission tomography. *JAMA Neurol*. 2014;71(10):1282-9. doi:10.1001/jamaneurol.2014.1358
8. Ovod V, Ramsey KN, Mawuenyega KG, et al. Amyloid beta concentrations and stable isotope labeling kinetics of human plasma specific to central nervous system amyloidosis. *Alzheimers Dement*. 2017;13(8):841-849. doi:10.1016/j.jalz.2017.06.2266
9. Schindler SE, Bollinger JG, Ovod V, et al. High-precision plasma beta-amyloid 42/40 predicts current and future brain amyloidosis. *Neurology*. 2019;93(17):e1647-e1659. doi:10.1212/WNL.0000000000008081
10. Barthelemy NR, Horie K, Sato C, Bateman RJ. Blood plasma phosphorylated-tau isoforms track CNS change in Alzheimer's disease. *J Exp Med*. 2020;217(11)doi:10.1084/jem.20200861
11. Janelidze S, Barthelemy NR, He Y, Bateman RJ, Hansson O. Mitigating the Associations of Kidney Dysfunction With Blood Biomarkers of Alzheimer Disease by Using Phosphorylated Tau to Total Tau Ratios. *JAMA Neurol*. 2023;80(5):516-522. doi:10.1001/jamaneurol.2023.0199
12. Janelidze S, Palmqvist S, Leuzy A, et al. Detecting amyloid positivity in early Alzheimer's disease using combinations of plasma Abeta42/Abeta40 and p-tau. *Alzheimers Dement*. 2022;18(2):283-293. doi:10.1002/alz.12395
13. Palmqvist S, Tideman P, Cullen N, et al. Prediction of future Alzheimer's disease dementia using plasma phospho-tau combined with other accessible measures. *Nat Med*. 2021;27(6):1034-1042. doi:10.1038/s41591-021-01348-z
14. Ashton NJ, Janelidze S, Mattsson-Carlgren N, et al. Differential roles of Abeta42/40, p-tau231 and p-tau217 for Alzheimer's trial selection and disease monitoring. *Nat Med*. 2022;28(12):2555-2562. doi:10.1038/s41591-022-02074-w
15. Pereira JB, Janelidze S, Smith R, et al. Plasma GFAP is an early marker of amyloid-beta but not tau pathology in Alzheimer's disease. *Brain*. 2021;doi:10.1093/brain/awab223
16. Gobom J, Parnetti L, Rosa-Neto P, et al. Validation of the LUMIPULSE automated immunoassay for the measurement of core AD biomarkers in cerebrospinal fluid. *Clin Chem Lab Med*. 2022;60(2):207-219. doi:10.1515/cclm-2021-0651
17. Horie K, Salvado G, Barthelemy NR, et al. CSF MTBR-tau243 is a specific biomarker of tau tangle pathology in Alzheimer's disease. *Nat Med*. 2023;29(8):1954-1963. doi:10.1038/s41591-023-02443-z
18. Palmqvist S, Rossi M, Hall S, et al. Cognitive effects of Lewy body pathology in clinically unimpaired individuals. *Nat Med*. 2023;29(8):1971-1978. doi:10.1038/s41591-023-02450-0
19. Quadalti C, Palmqvist S, Hall S, et al. Clinical effects of Lewy body pathology in cognitively impaired individuals. *Nat Med*. 2023;29(8):1964-1970. doi:10.1038/s41591-023-02449-7
20. Lundqvist R, Lilja J, Thomas BA, et al. Implementation and validation of an adaptive template registration method for 18F-flutemetamol imaging data. *J Nucl Med*. 2013;54(8):1472-8. doi:10.2967/jnumed.112.115006
21. Klunk WE, Koeppe RA, Price JC, et al. The Centiloid Project: standardizing quantitative amyloid plaque estimation by PET. *Alzheimers Dement*. 2015;11(1):1-15 e1-4. doi:10.1016/j.jalz.2014.07.003
22. Rafii MS, Sperling RA, Donohue MC, et al. The AHEAD 3-45 Study: Design of a prevention trial for Alzheimer's disease. *Alzheimers Dement*. 2023;19(4):1227-1233. doi:10.1002/alz.12748
23. Amadoru S, Dore V, McLean CA, et al. Comparison of amyloid PET measured in Centiloid units with neuropathological findings in Alzheimer's disease. *Alzheimers Res Ther*. 2020;12(1):22. doi:10.1186/s13195-020-00587-5
24. La Joie R, Ayakta N, Seeley WW, et al. Multisite study of the relationships between antemortem [(11)C]PIB-PET Centiloid values and postmortem measures of Alzheimer's disease neuropathology. *Alzheimers Dement*. 2019;15(2):205-216. doi:10.1016/j.jalz.2018.09.001
25. Bollack A, Collij LE, Garcia DV, et al. Investigating reliable amyloid accumulation in Centiloids: Results from the AMYPAD Prognostic and Natural History Study. *Alzheimers Dement*. 2024;doi:10.1002/alz.13761

© 2024 Janelidze S et al. *JAMA Neurology*.
