# Supplementary material for: Plasma Phosphorylated Tau 217 and Aβ42/40 to Predict Early Brain Aβ Accumulation in People Without Cognitive Impairment
Source: JAMA Neurol. 2024 Jul 28;81(9):947–57. doi: 10.1001/jamaneurol.2024.2619 (PMC11284634; doi:10.1001/jamaneurol.2024.2619)
Supplement: Supplement 2. — Data Sharing Statement. [file jamaneurol-e242619-s002.pdf]

Data Sharing Statement

Janelidze. Plasma Phosphorylated Tau 217 and Aβ42/40 to Predict Early Brain Aβ Accumulation in People Without Cognitive Impairment. *JAMA Neurol*. Published July 28, 2024. doi:10.1001/jamaneurol.2024.2619

Data
Data available: No

Additional Information
Explanation for why data not available: Anonymized data will be shared by request from a qualified academic investigator for the sole purpose of replicating procedures and results presented in the article and as long as data transfer is in agreement with EU legislation on the general data protection regulation and decisions by the Ethical Review Board of Sweden and Region Skåne, which should be regulated in a material transfer agreement.
